# Integrated morphological, molecular, histological, and antimicrobial analysis of the leather leaf slug *Eleutherocaulis alte* from Assiut Governorate, Egypt

**DOI:** 10.1038/s41598-025-32703-6

**Published:** 2026-01-06

**Authors:** Safaa M. Ali, Torkia A. Mohammed, Shimaa H. Salem, Hayam A. Saber, Asmaa R. Abdel-Malek

**Affiliations:** 1 Zoology and Entomology Department, Faculty of Science, Assiut University, Assiut, 71526 Egypt; 2 Botany and Microbiology Department, Faculty of Science, Assiut University, Assiut, 71526 Egypt

**Keywords:** Leatherleaf slug, Eleutherocaulis alte, Veronicellidae, Histological analysis, Mucus-secretory cells, Terrestrial slugs, Biochemistry, Biological techniques, Biotechnology, Microbiology

## Abstract

The leatherleaf slug *Eleutherocaulis alte* from Assiut Governorate, Egypt, was investigated using an integrative approach combining morphological, molecular, histological, and bioactivity analyses. Morphological characterization revealed a dorsoventrally flattened body with a brown dorsal surface marked by a pale median line, dark spots, and a narrow central foot. Mitochondrial cytochrome c oxidase I (COI) gene sequencing confirmed its identity as *E. alte*, showing 98.23% similarity to *Laevicaulis alte*, and the sequence was deposited in GenBank (OR162029). Scanning electron microscopy demonstrated porous mucus-secreting surfaces essential for locomotion and adhesion, while histological examination revealed distinct secretory cell types within the epidermal and subepidermal layers, including a suprapedal gland producing mixed acidic and neutral mucopolysaccharides. Bioactivity assays indicated that the crude mucus exhibited potent antimicrobial activity, particularly against *Bacillus subtilis* and *Candida albicans*, with minimum inhibitory concentrations of 7.8 µg/mL and 3.9 µg/mL, respectively. The crude mucus showed significantly greater antimicrobial activity against Gram-positive and Gram-negative bacteria as well as *C. albicans* compared to the corresponding positive controls (gentamicin or fluconazole; *P* < 0.05 − 0.001). However, it exhibited no inhibitory effect against *Aspergillus niger*, collectively, these findings provide novel taxonomic, anatomical, and biomedical insights into *E. alte*, and highlight its mucus as a promising natural source of antimicrobial agents.

**Supplementary Information:**

The online version contains supplementary material available at 10.1038/s41598-025-32703-6.

## Introduction

Terrestrial slugs play a crucial role in ecosystems by processing dead organic matter, redistributing nutrients, and forming the dietary basis for many animals^1^. Despite their ecological and economic importance, they pose a significant threat to agriculture worldwide as they infesting ornamental plant nurseries and crop fields, where they cause significant economic losses by consuming seeds and seedlings and damaging various plant parts^2,3^. Additionally, they contaminate agricultural products with their bodies, feces, or slime that also reduces the quality and commercial value^4^. Their impact has increased in recent years due to their adaptability, polyphagous feeding habits, and unintentional spread through the international transport of agricultural products^5,6^. Among these, slugs of the family Veronicellidae (order Systellommatophora) are notable for their dorsoventrally flattened bodies and high reproductive capacity, facilitating their widespread distribution across agricultural regions^7,8^.

Recent surveys in northeastern Africa have documented three species of *Laevicaulis* in agricultural fields: *Laevicaulis alte* in Giza Governorate^9,10^, *Laevicaulis stuhlmanni aegypti*, a new subspecies described from Cairo^4^, and *Laevicaulis striatus*, reported from Libya^11^. These species are differentiated by features such as dorsal coloration and genital morphology^12,13^. *L. alte*, commonly called the “tropical leatherleaf slug,” is characterized by a leathery, granulated dorsal mantle interrupted by a pale median stripe. Though native to Africa^14^, *L. alte* has been introduced to Australia, southern Asia, and the Pacific Islands^15^. In Egypt, it was first recorded in 2018 on ornamental plants in Abo Rawash, Giza^9^.

Terrestrial gastropods are known for their copious mucus production, which supports adhesion, locomotion, reproduction, defense, and desiccation resistance^16,17^. This functional diversity reflects underlying differences in secretory gland types. Gastropod epithelia typically harbor multiple types of secretory cells, including mucus, channel, and calcified cells, previously termed unicellular or subepithelial glands^17^. The number, distribution, and chemical composition of these cells vary both between and within species^18^. For example, two to three gland types have been documented in *Arion ater*^19,20^, while four ventral glands have been described in *Arion rufus*^21^.

Among the most studied slug species, *Agriolimax* and *Arion* produce a viscoelastic dorsal slime used for predator deterrence, which solidifies within minutes and differs structurally and functionally from locomotory mucus^17,22^. This adhesive slime contains unique proteins that trigger gel stiffening^23^. The tropical Veronicellidae genus *Veronicella* exhibits a similar defense mechanism, although it differs taxonomically and histologically. Veronicellids possess up to 11 gland types, with only 2–3 discharging mucus onto the dorsal surface. Their dorsal mucus is rich in carboxylated mucopolysaccharides, while ventral pedal mucus, produced by transverse-ridged glands and a short suprapedal gland, contains a mix of neutral and weakly acidic mucopolysaccharides, as well as proteins.

Snail and slug mucus is composed primarily of water (90–99.7%) and a small fraction of bioactive substances, including glycoproteins, enzymes, antimicrobial peptides, glycosaminoglycans (e.g., hyaluronic acid), and metal ions essential for gel formation^24^. Additional components such as elastin, collagen, allantoin, and antioxidant enzymes (e.g., GST, SOD) contribute to its healing, antimicrobial, and anti-inflammatory properties. These features have made mucus an increasingly valuable resource in the pharmaceutical and cosmetic industries^24–27^. Some compounds have also shown neuroprotective activity, highlighting their broader therapeutic potential^28^.

Considering these findings, the present study employs an integrative framework to establish a comprehensive baseline characterization of *E. alte* collected from Assiut Governorate, Egypt. While the core focus lies in elucidating the species’ anatomical, molecular, and histological features, the antimicrobial assessment of crude mucus was included to provide preliminary insight into the potential biomedical relevance of this species. These antimicrobial data are intended as a foundational step toward more advanced biochemical and pharmacological investigations. Overall, this multidisciplinary approach contributes novel insights into the taxonomy, physiology, and applied biomedical potential of this understudied terrestrial slug species.

## Materials and methods

### Collection and morphological identification of slugs

A total of 128 adult slugs, measuring 4 ± 2 cm in length and weighing about 2 ± 0.5 g, were manually collected during the early morning hours from various sites within the Assiut University Farm. These included ornamental plant nurseries and a range of humid agricultural fields, such as those cultivating fruits and vegetables. The collection period extended from September 2021 to May 2024. Following collection, the slugs were transferred to the laboratory of the Department of Zoology and Entomology under ambient conditions (temperature: 30–35 °C). Specimens were maintained for approximately two weeks in plastic containers (radius = 11 cm, height = 10 cm) containing moist soil. They were fed fresh and healthy lettuce leaves, commercially purchased from a local market, with uneaten food removed daily to maintain hygiene (Fig. S1). Prior to morphological identification, the anesthetization of slugs was carried out with particular care to prevent excessive withdrawal of tentacles and self-coiling, which could obscure diagnostic external features and complicate dissection. To ensure progressive relaxation, slugs were first gradually exposed to the vapors of 70% ethanol until partial immobilization was observed. They were then immersed completely in tap water for approximately one hour to achieve full relaxation before fixation. This gradual method allowed maximum muscle relaxation while minimizing stress responses and physical distortion.

Following complete relaxation, specimens were fixed in Kahl’s fixative for 24 h^29^, then preserved in 70% ethanol until further examination. Throughout handling, specimens were monitored for signs of distress such as excessive mucus secretion, loss of body turgor, or uncoordinated movement. Any individuals exhibiting persistent distress were immediately euthanized using the same protocol. No slugs were maintained beyond humane endpoints. Identification was performed based on external morphological characteristics following the criteria outlined by Ali^4^, Ali and Robinson^10^, and Ali et al.^30^. Key diagnostic features included the shape of the anus, the distance between the female genital opening and the foot groove, and the ratio of foot length to total body length. Additionally, anatomical analysis of the genital system was conducted on 20 specimens, which were dissected according to the method of Bishop^31^, with minor modifications. Relaxed and fixed slugs were incised along the left hyponotum to expose internal organs. Both male and female genital structures were examined and photographed for documentation. All procedures were performed in accordance with the ethical standards of Assiut University, national regulations governing the care and use of animals in research, and the international guidelines outlined by the OIE (World Organization for Animal Health). This study was conducted and is reported in accordance with the ARRIVE guidelines for the ethical reporting of animal research.

## Molecular identification

### DNA extraction, PCR amplification of rDNA, and sequencing

Genomic DNA was extracted from preserved samples (*n* = 2) in accordance with the manufacturer’s instructions, utilizing the QIAamp DNA Mini kit (Qiagen, Hilden, Germany). The partial mitochondrial cytochrome c oxidase I (COI) gene was amplified using the forward primer LCO1490 (5′-GGT CAA CAA ATC ATA AAG ATA TTG G-3′) and the reverse primer HCO2198 (5′-TAA ACT TCA GGG TGA CCA AAA AAT CA-3′), following the methodology described by Vrijenhoek^32^. Polymerase chain reaction (PCR) was performed in a total volume of 50 µL containing 25 µL of 2× PCR Master Mix, 1 µL of each primer (forward and reverse), 22 µL of molecular grade water, and 1 µL of genomic DNA. The amplification protocol consisted of an initial denaturation at 94 °C for 5 min, followed by 30 cycles of denaturation at 94 °C for 60 s, annealing at 49 °C for 60 s, and extension at 72 °C for 60 s. A final extension step was performed at 72 °C for 10 min. The amplified products were verified by electrophoresis on a 1.5% agarose gel containing ethidium bromide and visualized under UV illumination. A 100 bp DNA ladder was used for fragment size estimation. The purified amplicons were sequenced bidirectionally by Macrogen Inc. (Seoul, South Korea). The final COI sequence was submitted to the National Center for Biotechnology Information (NCBI) to obtain an accession number.

### Sequence alignment and phylogenetic analysis

The contiguous sequence of the sample was constructed using DNASTAR (version 5.05). For the phylogenetic analysis, a dataset comprising 16 COI gene sequences was employed, including one sequence from the current study’s *Eleutherocaulis* sample, 14 sequences of the most closely related species retrieved from GenBank, and a sequence from *Panagrolaimus labiatus* isolate SN14_I, which was used as an outgroup. Sequences were aligned using MAFFT (version 6.861b) with default settings^33^. Alignment gaps and parsimony-uninformative characters were optimized using BMGE^34^. Maximum-likelihood (ML) and maximum parsimony (MP) phylogenetic analyses were performed with MEGA X version 10.2.6^35^. The reliability of the most parsimonious trees was assessed through 1000 bootstrap replications^36^. The optimal nucleotide substitution model for ML analysis was determined using the Akaike Information Criterion (AIC), as implemented in Modeltest 3.7^37^. The resulting phylogenetic tree was visualized and edited in MEGA X^35^, and the final figure was saved as a TIF file^38^.

### Scanning electron microscopy study

Specimens of *E. alte* were fixed for 2 h in 4% glutaraldehyde in cacodylate buffer with pH = 7.4, washed in the same buffer. Then they were post-fixed in 2% Osmic acid and washed again in cacodylate buffer, dehydrated in ascending concentrations of ethanol. Then, the prepared samples were examined under a JSM 5400 LV (SEM) at the Electron Microscope Unit, Assiut University.

### Histological and histochemical preparations

Five relaxed specimens were fixed in Kahl’s solution^29^ for 24 h. Each specimen was manually divided into three regions, anterior, mid, and posterior, and transverse paraffin sections of 6 μm thickness were prepared from each region to examine potential differences across the body wall. Histological analysis focused on the detailed structure of the mucus secretory cells in various body surfaces, including the dorsal (notum), lateral (perinotum), and ventral regions (comprising the foot sole, foot grooves, and adjacent hyponota), as well as the suprapedal gland.

Histochemical investigations were performed following Carleton et al.^39^ using a series of specific stains: hematoxylin and eosin (H&E) for general histological examination; Milligan trichrome for collagen; periodic acid–Schiff (PAS) reaction (1% freshly prepared periodic acid and Schiff reagent, 30 min incubation) for polysaccharides; Alcian blue at pH 2.5 and pH 1.0 (10 min incubation) for detection of acidic and sulfated mucopolysaccharides, respectively; combined Alcian blue–PAS for differentiation between neutral and acidic mucopolysaccharides; toluidine blue (1% in 30% ethanol, 10 min incubation) for identifying metachromatic (carboxylated and sulfated) and orthochromatic structures; and mercury bromophenol blue (1% in distilled water, 30 min incubation) for total protein detection.

All stained sections were examined using a Leitz Dialux 20 light microscope and imaged with a Canon PowerShot A95 digital camera at ×20 and ×40 magnification.

### Terminology of the mucous secretory cells

The nomenclature of mucous secretory cells follows the standardized terminology for gastropod secretory glands as described by Smith^16^ and Greistorfer et al.^40^. Each glandular cell is designated by a three-letter code, structured as follows: the first letter represents the initial of the genus name; the second component is a sequential number; and the third is an abbreviation indicating the anatomical region of the body in which the cell is located “d” for dorsal, “l” for lateral, and “v” for ventral.

### Mucus collection and preparation

A total of 83 slugs were initially washed with distilled water and 70% ethanol to remove surface contaminants. To induce mucus secretion, a stimulating solution composed of 5% vinegar and salt was applied. The slugs were then placed in a clean Petri dish (Fig. S2) to facilitate mucus production. The secreted mucus was subsequently collected in sterile falcon tubes, lyophilized into a white powder, and stored at −20 °C until further use (Fig. S2). The powder was then processed as crude mucus (CM).

### Antimicrobial assay

#### Microbial strains used

A total of six bacterial strains were employed in this study: *Escherichia coli* ATCC 8739, *Salmonella typhi* ATCC 6539, *Klebsiella pneumoniae* ATCC 13,883, *Bacillus subtilis* ATCC 6633, *Staphylococcus aureus* ATCC 6538, and *Staphylococcus epidermidis* ATCC 12,228. In addition, two fungal strains were tested: *Candida albicans* ATCC 10,221 and a clinical isolate of *Aspergillus niger*, which was previously isolated from sputum samples of patients at Assiut University Hospital and kindly provided by the Assiut University Mycological Centre (AUMC, Assiut, Egypt).

### Agar well diffusion assay

The antimicrobial activity of the mucus was evaluated using the agar well diffusion method on Luria-Bertani (LB) agar and Sabouraud dextrose agar (SDA) plates for bacterial and fungal strains, respectively^41^. Bacterial suspensions were adjusted to a 0.5 McFarland standard (approximately 1 × 10⁸ CFU/mL), while fungal spore suspensions were prepared at a concentration of 1 × 10⁶ CFU/mL. One milliliter of each standardized inoculum was evenly spread onto the surface of sterile LB or SDA plates. Sterile cork borers were used to create wells of 8 mm diameter in the inoculated agar. The lyophilized crude mucus powder was dissolved in dimethyl sulfoxide (DMSO) to prepare a 10 mg/mL of the mucus, which served as the initial screening concentration for evaluating the antimicrobial potential of the crude mucus. Each well was filled with 100 µL of mucus. Gentamicin (10 mg/mL) was used as the positive control for bacterial strains, while fluconazole (10 mg/mL) served as the positive control for fungal strains. 10% of DMSO solution was used as the negative control. All assays were conducted in triplicate. Antimicrobial activity was assessed by measuring the diameter of the inhibition zones around each well, and the average values were calculated.

### Determination of MIC, MBC, and MFC values

To evaluate the antimicrobial efficacy of the crude mucus, minimum inhibitory concentration (MIC), minimum bactericidal concentration (MBC), and minimum fungicidal concentration (MFC) values were determined using the broth microdilution method, as described by Abdel-Malek et al.^41^, and in accordance with the Clinical and Laboratory Standards Institute (CLSI) guidelines^42,43^. Briefly, twofold serial dilutions of the crude mucus in the appropriate test medium, Mueller-Hinton broth (MHB) for bacterial strains, and Sabouraud dextrose broth (SDB) for *C. albicans*, were placed in a 96-well round bottom plate at concentrations ranging from 1000 to 1.95 µg/mL.

Microbial inocula were prepared using the direct colony suspension method. Fresh colonies were suspended in sterile 0.85% saline and adjusted to 0.5 McFarland standard, corresponding to 1 × 10⁸ CFU/mL for bacteria and 1 × 10⁶ CFU/mL for *C. albicans*. Suspensions were subsequently diluted in the corresponding test medium to achieve final inoculum densities of 5 × 10⁵ CFU/mL per well for bacteria, 0.5 × 10³–2.5 × 10³ CFU/mL for *C. albicans*, as recommended by CLSI. Each well received 100 µL of the prepared mucus dilution and 100 µL of microbial inoculum, giving a final volume of 200 µL.

Bacterial plates were incubated at 35 ± 2 °C for 16–20 h, whereas yeast plates were incubated at 35 ± 2 °C for 24–48 h. Sterility, growth, and solvent controls were included to verify that antimicrobial activity was solely attributable to the crude mucus.

The MIC is defined as the lowest concentration of crude mucus that completely inhibited visible microbial growth when examined by the unaided eye^42^. To determine the MBC and MFC, aliquots from wells showing no visible turbidity were aseptically subcultured onto Mueller-Hinton agar (MHA) for bacterial strains and SDA for fungal strains. The plates were incubated under the same conditions as those used for MIC determination. The MBC and MFC were defined as the lowest concentrations that yielded no visible colony growth on the agar surface, corresponding to a ≥ 99.9% reduction in viable cell counts relative to the initial inoculum. According to established criteria, the bactericidal and fungicidal endpoints represent the concentrations required to achieve 99.9% and 98–99.9% microbial killing, respectively^44,45^.

### Statistical analysis

All antimicrobial assays were performed in triplicate, and results are expressed as mean ± standard deviation (SD). Comparative analyses between the inhibition zones produced by the crude mucus and those of the reference antimicrobial agents (gentamicin and fluconazole) were conducted using independent *t*-tests. Statistical analyses were performed using standard procedures, and a *P*-value < 0.05 was considered statistically significant.

## Results

### Morphological description

**Fig. 1:**
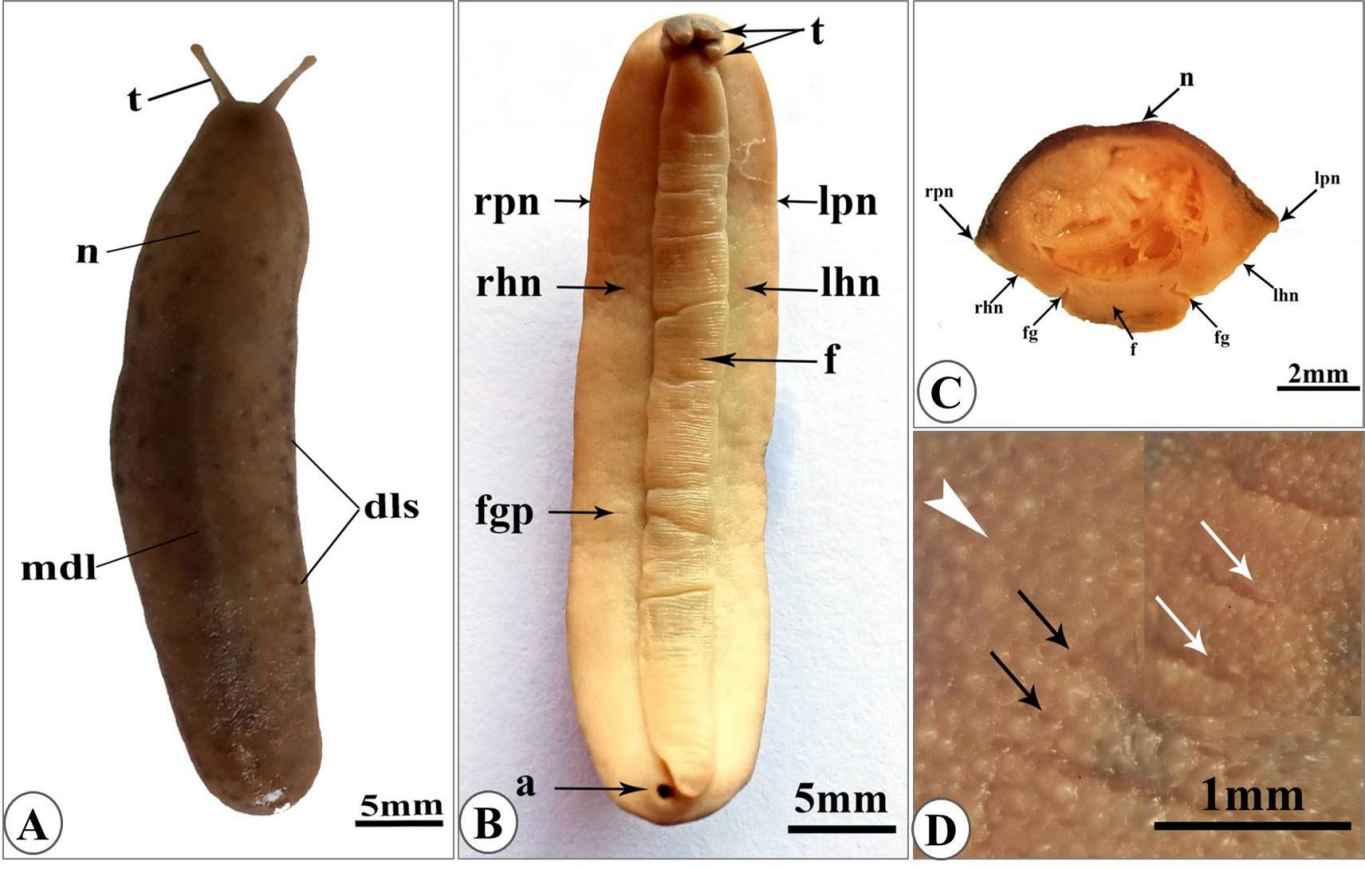
Photographs of the slug *“Eletherocaulis alte”* **(A)** dorsal view of live specimen showing mantle covers all the dorsal surface (notum, n) with protruded anterior pair of tentacles (t). The notum appeared in brown interrupted by a pale median dorsal line (mdl) and mottled with dark spots arranged as dorsolateral stripes (dls) or scattered on the entire surface. **(B)** The ventral view of a fixed specimen showing the central narrow foot (f) surrounded by the lateral right hyponotum (rhn) and left hyponotum (lhn). The female genital pore (fgp) and the anus (a) opened in the right hyponotum. **(C)** hand section of the specimen showing the dorsoventrally flattened body with dorsal notum wrapping ventrally as hyponotum and tapered laterally giving right perinotum (rpn) and left perinotum (lpn), the foot separated from the surrounding hyponota by foot groove (fg). **(D)** enlarged dorsal notum with granulated appearance (arrowhead), minute pores (black arrows), and transvers forrows (white arrows).

The body of *E. alte* (leatherleaf slugs) is dorsoventrally flattened and tapering laterally. Mantle covers the entire dorsal surface (notum) and wraps beneath the animal to form the ventral hyponotum. These two surfaces are connected by two lateral tapered ridges named perinotum (Fig. 1). The dorsal surface appears brown, interrupted by a pale median line and mottled (punctuated) with dark spots. These spots are either arranged as dorsolateral stripes or scattered on the entire surface of the notum (Fig. 1A), which also possesses a granular appearance with several minute pores and transverse furrows (Fig. 1Aand D). The ventral surface is yellowish, with the foot centrally appeared and the hyponota on the two lateral sides, the foot sole appears with prominent transverse ridges that are obvious in both live (Fig. 1A) and fixed specimens (Fig. 1B). Foot restricted to a narrow region (narrower than the ventral body width) and separated from the surrounding hyponota with a foot groove (Fig. 1C).

**Fig. 2:**
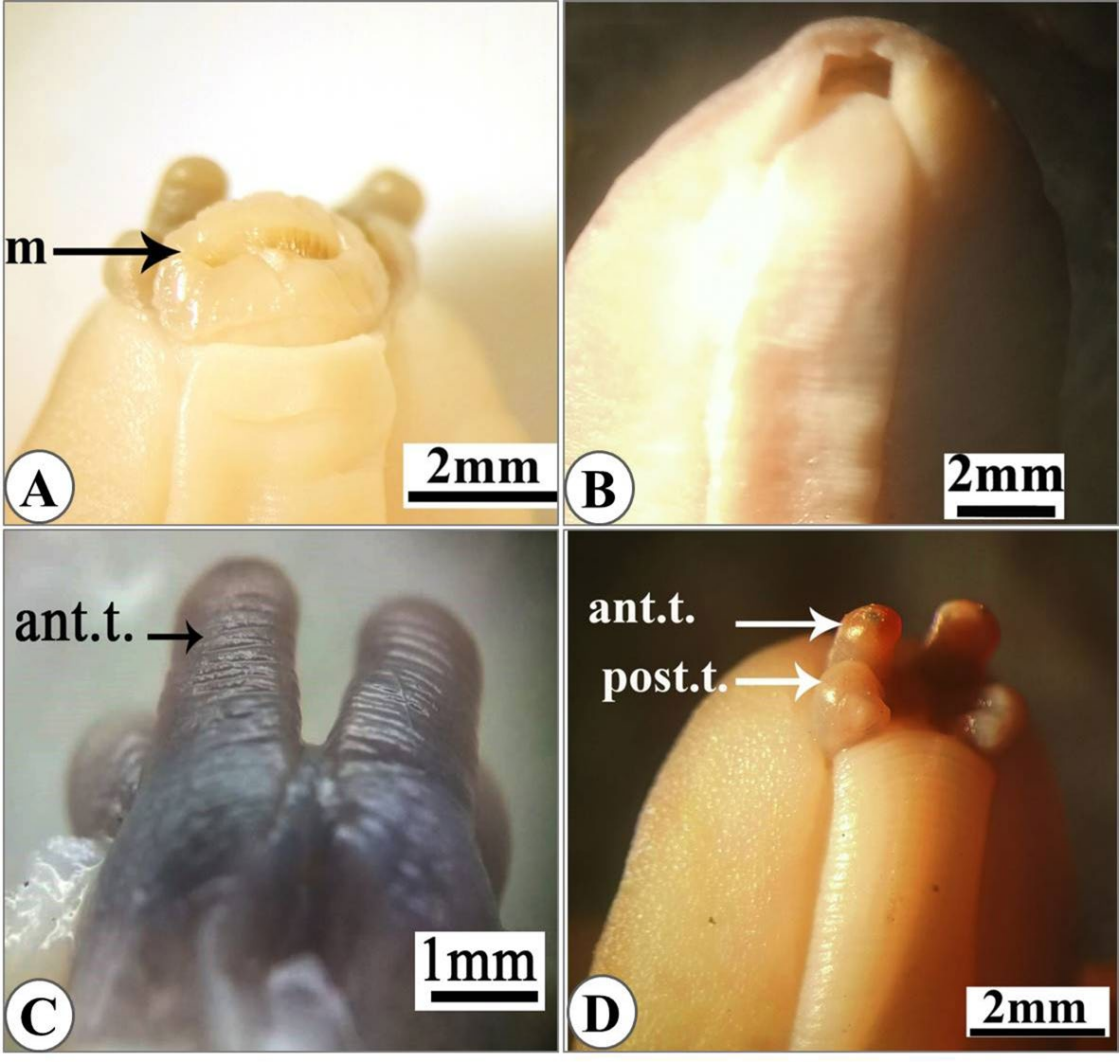
Photographs of the anterior region of the specimens. **(A)** with protruding mouth (m). **(B)** completely withdrawal mouth. **(C)** the anterior annulated tentacles (ant.t.). **(D)** bilobed posterior tentacles (post.t.).

**Fig. 3:**
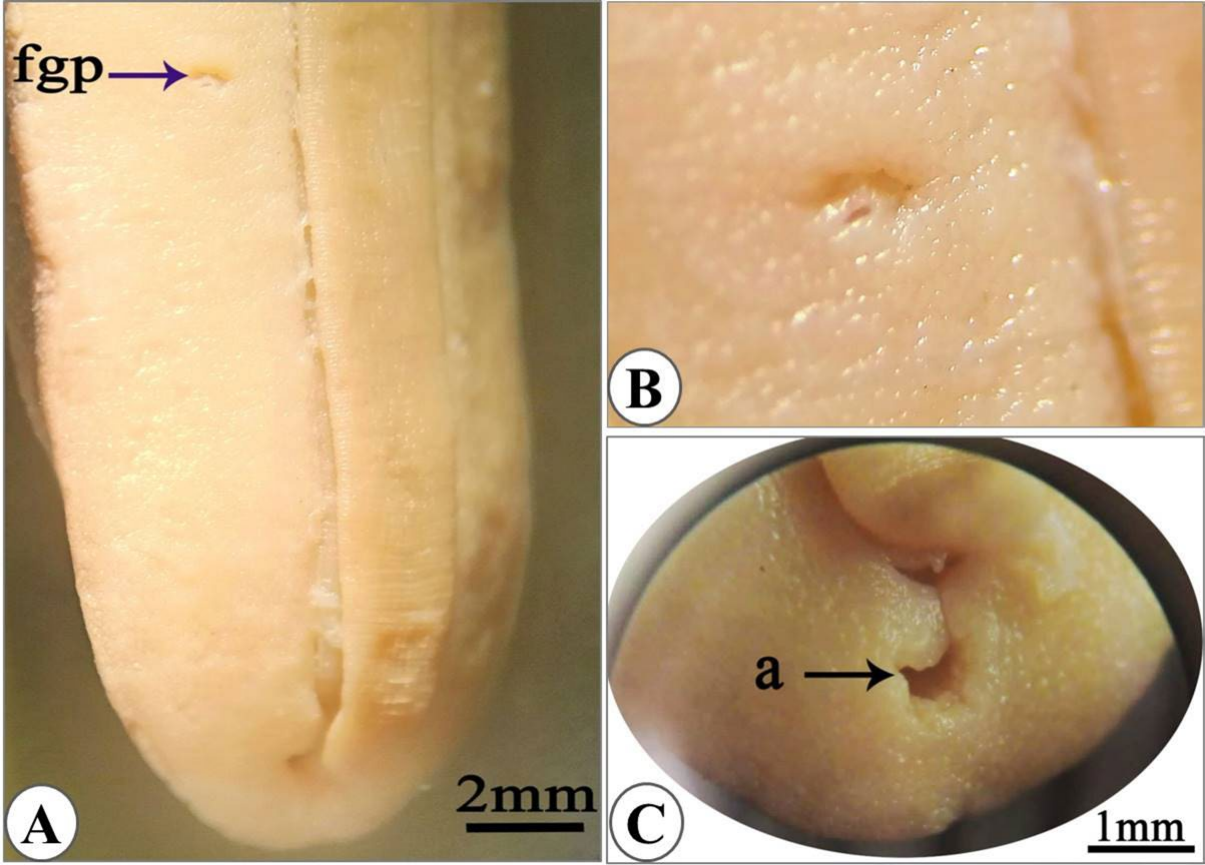
**(A)** Ventral view of the specimen with female genital pore (fgp) closer to the foot. (**B)** enlarged female genital pore. (**C)** anus with crescentic shape opening.

The mouth protrudes anteriorly from the ventral side in relaxed specimens (Fig. 2A) or completely withdraws in the contracted specimen (Fig. 2B). The ocular pair of tentacles (which pear the eyes) are annulated (Fig. 2C) and the olfactory pairs are bilobed (Fig. 2D). Genital pore, located on the right hyponotum of the animal closer to the foot than the outer edge of body (less than 1\4 hyponotal width) at the last third of hyponotal length (Fig. 3A) or near the middle of the right hyponotum (Fig. 3B). Breathing pore not visible. Anal slit, clearly visible with naked eye extending behind the right side of the foot. The anus is oval with superficial opening and appears as transverse crescentic slit (Fig. 3C).

### Anatomy of genital system

**Fig. 4:**
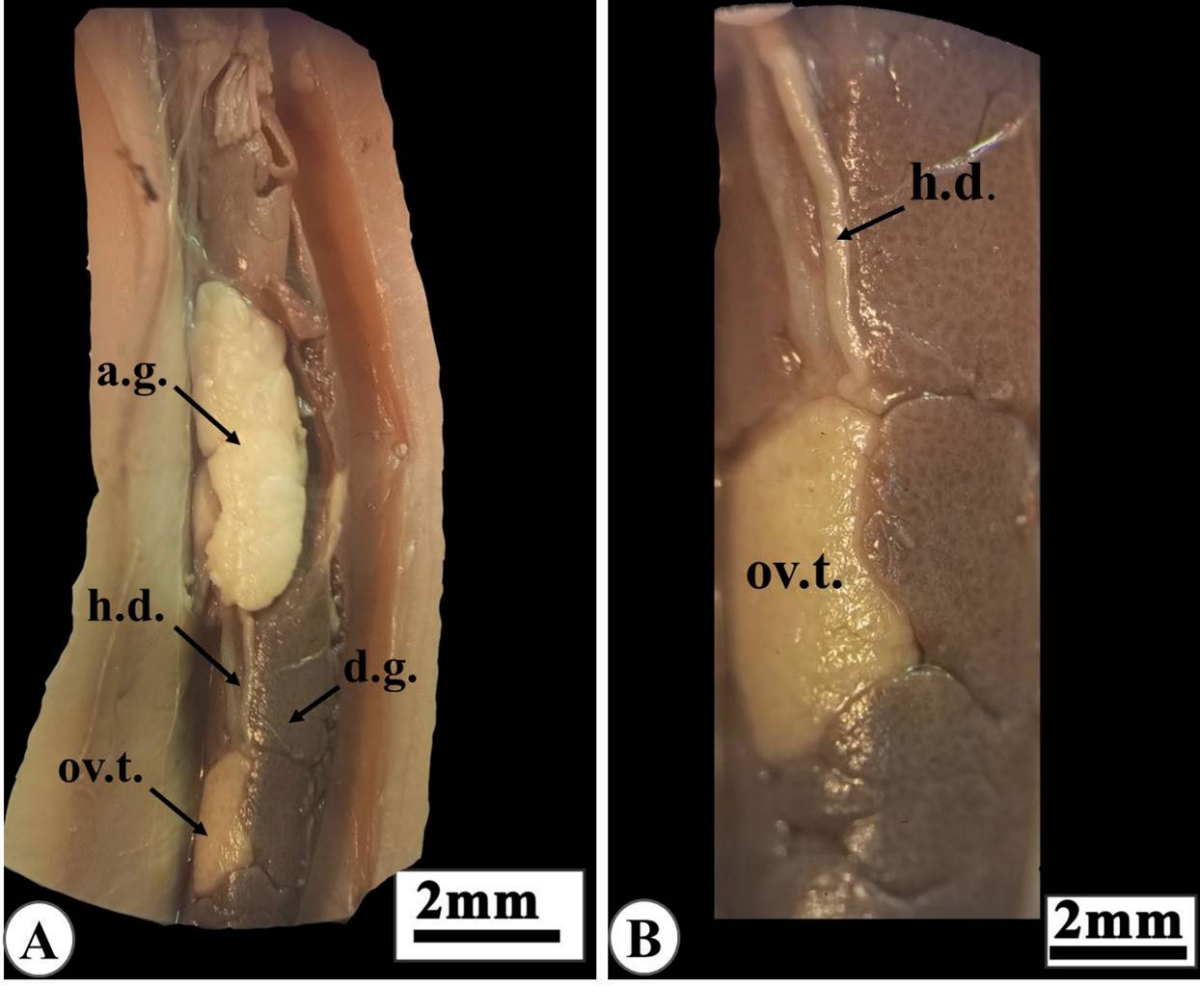
Photographs of dissected slug “*Eletherocaulis alte*” showing anatomy of the reproductive system: **(A)** the female reproductive organs, including ovotestis (ov.t.) embedded between the digestive gland lobes and the hermaphrodite duct (h.d.) that connected to the albumin gland (a.g.). **(B)** Enlarged ovotestis with its hermaphrodite duct.

**Fig. 5:**
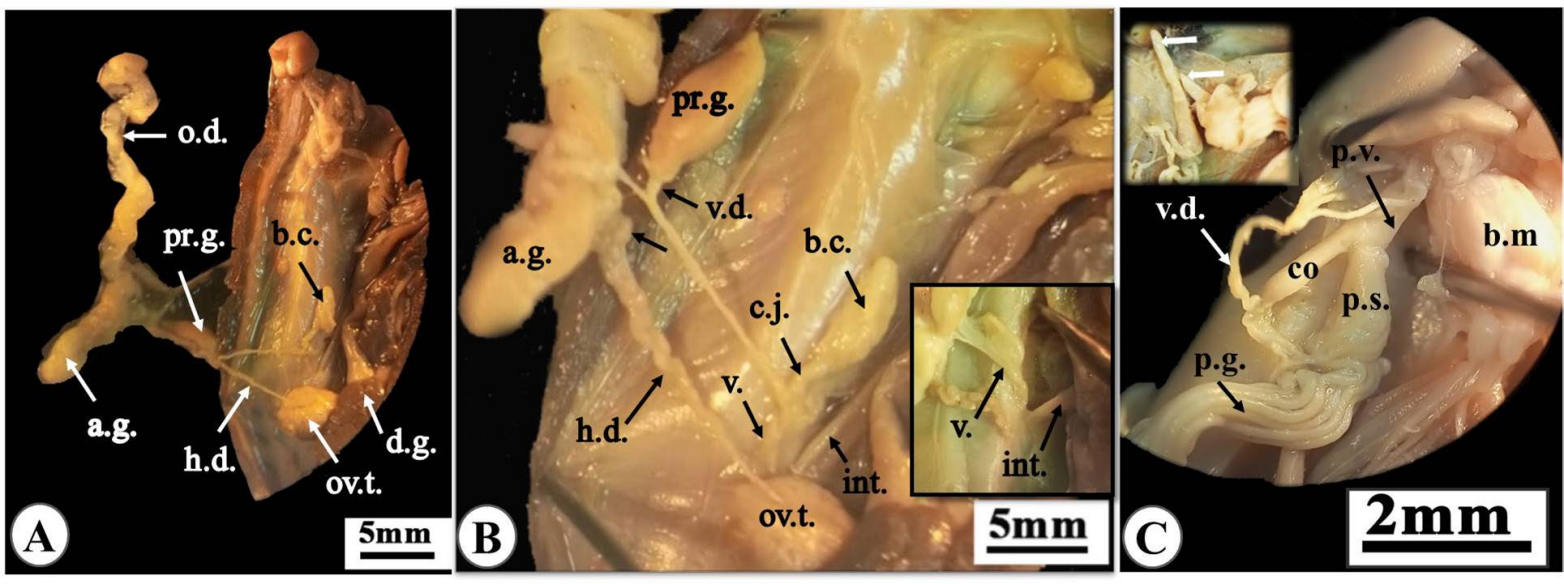
Photographs of **(A)** dissected hermaphroditic genital system and **(B)** magnified portion from it showing: the ovotestis (ov.t.) leads to the hermaphrodite duct (h.d) which connected to the carrefour (c.), along oviduct (o.d.) receiving the albumen gland (a.g.) and leads to the vagina (v) that opens in the female genital opening on the right hyponotum. The bursa copulatrix (b.c.) connected to the vagina (v.) by a short canalis junctor (c.j.). The male genital tract begins with the vas deferens (v.d.) which receives the prostate gland (p.g.) and gives of the canalis junctor, then enter the body wall beside the vagina. **(C)** The pineal complex at the right side of the buccal mass (b.m.) that consists of the penial verge (p.v.) with subdistal swelling, collar (co.), and the penial stimulator (p.s.), which is supported by a mucus pineal gland (p.g.). The white arrow refers to the apex of penial verge is taller than the penial stimulator.

Veronicellidae reproductive system is hermaphrodite. Ovotestis is located between the digestive gland lobes (Fig. 4A) and leads to the hermaphrodite duct (Fig. 4B). Then the duct leads to the fertilization chamber, which connects to the carrefour (the junction area between the male and female ducts). The female genital tract involves the oviduct connected with the albumen gland near the carrefour then lead to a muscular tube known as vagina that open in the right hyponotum in the female genital opening (Fig. 5A).

The male genital tract represented by the vas deferens that connected by the prostate gland close to the carrefour leading to the canalis junctor then enters the right body wall parallel to the vagina of the female tract (Fig. 5B). From this point it passes forward near a structure known as “Penial complex” that located at the right side of the buccal mass. This complex includes the intromittent penial verge and the stimulator of the penial and its gland that consists of a group of tubules. Both the stimulator and the verge are enclosed within a thin sheath supplied with retractor muscles. The vagina is connected to the bursa copulatrix by a pedicle. This bursa copulatrix is also connected to the male genital system by the canalis junctor (Fig. 5C).

### Molecular study and phylogenetic analysis

**Fig. 6:**
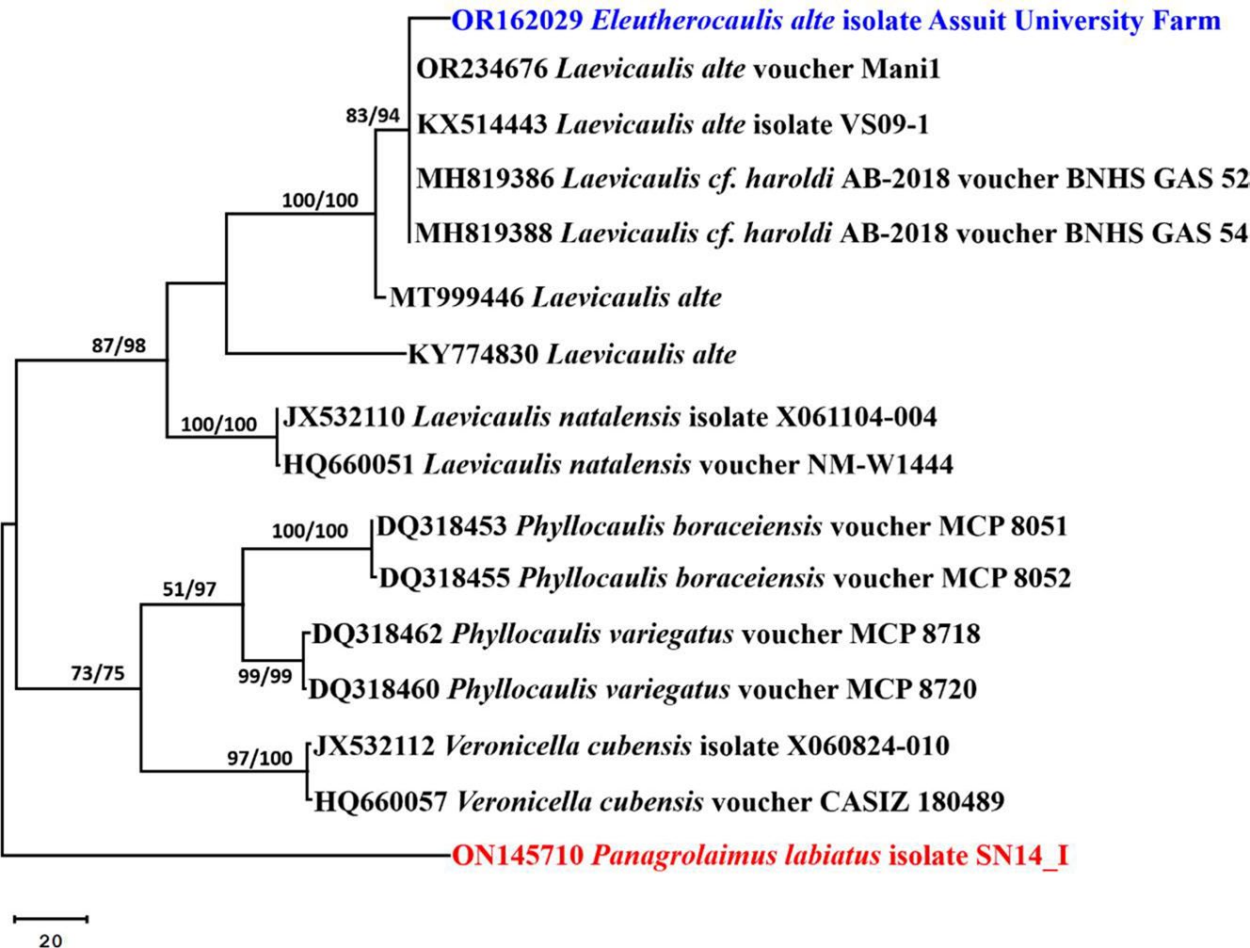
The most parsimonious phylogenetic tree obtained from a heuristic search (1,000 replications) of *Eleutherocaulis alte* isolate Assiut University Farm (in blue) compared with closely related COI sequences retrieved from GenBank. Bootstrap support values for ML/MP ≥ 50% are shown at the respective nodes. The tree is rooted to *Panagrolaimus labiatus* isolate SN14_I (in red) as the outgroup.

Based on a megablast search of NCBIs GenBank nucleotide database, the closest hits using COI sequence of *Eleutherocaulis* sample in this study are *Laevicaulis alte* isolate VS09-1 [(GenBank accession number KX514443; identities = 611/622 (98.23%); gaps = 0/622 (0%)]. The COI sequence of the sample in this study was compared to other similar species using a phylogenetic analysis. The COI data set comprised 16 sequences totaling 617 characters, of which 448 could be successfully aligned (with no gaps or N), 210 characters were recorded as variable, and 145 characters were rated as informative. The Maximum Parsimony method was used to create 10 trees in order to deduce the evolutionary history. The most parsimonious phylogenetic tree for all sites and parsimony-informative sites is shown in Fig. 6 with the highest log likelihood (−2814.50), consistency index of 0.752551, retention index of 0.861626, and composite index of 0.648418. In the phylogenetic tree, *Eleutherocaulis* sample in this study was clustered in a strong supported clade (83% ML/94% MP) with *Laevicaulis alte* samples. As a result, this strain is identified here as *E. alte*. The COI sequence of *E. alte* was uploaded to GenBank as OR162029.

### SEM analysis

**Fig. 7:**
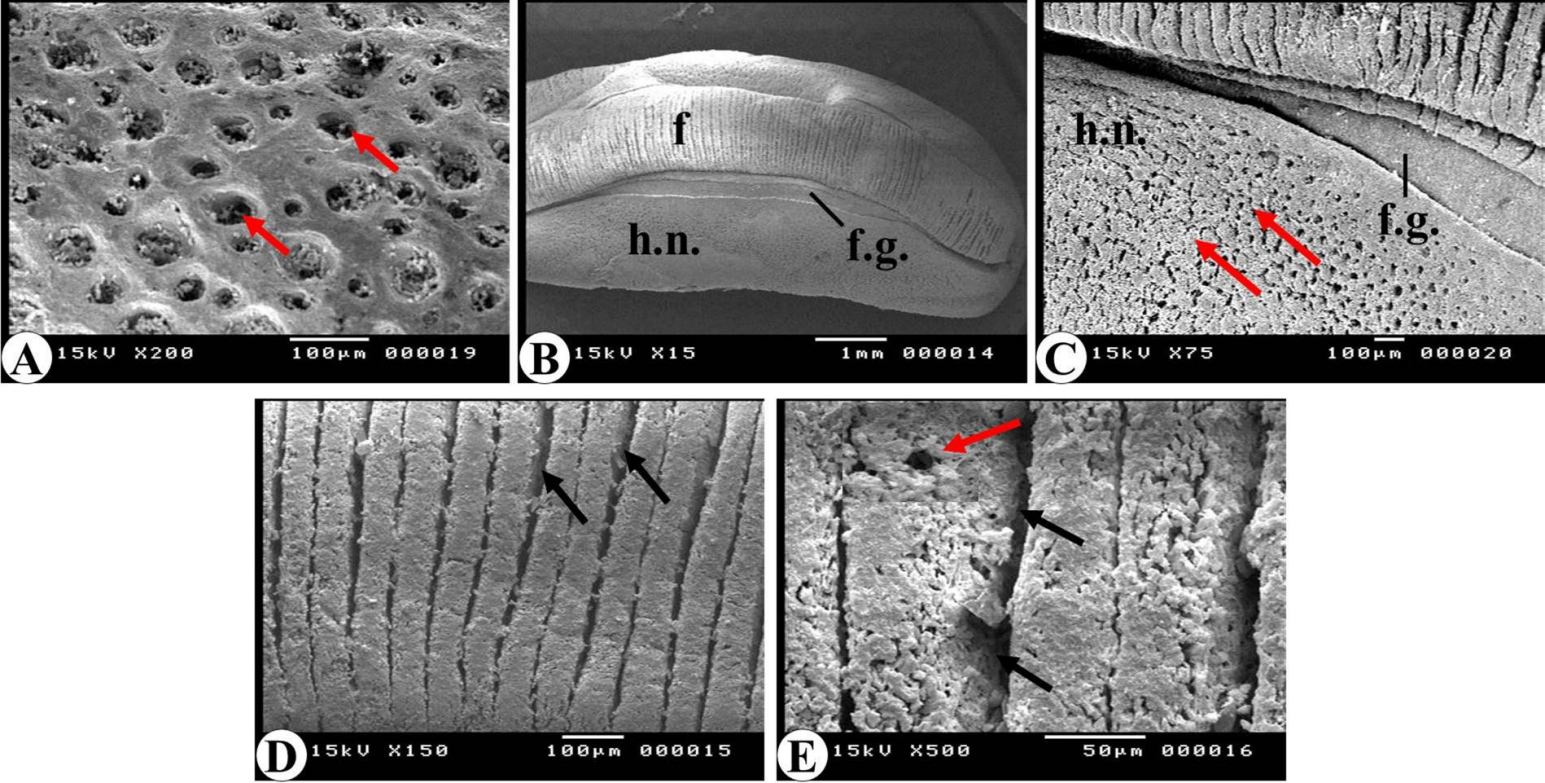
Scanning electron micrograph of the leatherleaf slug “*Eleutherocaulis alte”* showing: **(A)** the dorsal surface (notum) with large dorsal pores (white arrows). **(B)** the ventral surface of the specimen showing the central foot (f), foot groove (f.g.) and hyponota (h.n.). **(C)** the perforated surface of the hyponotum. **D and E)** the central foot with several transverse folds (black arrows) and pores (white arrows).

Scanning electron microscopy revealed that the external surface of the notum in *E. alte* is characterized by the presence of prominent pores (Fig. 7A), which serve as outlets for the secretion of dorsal mucus. The ventral surface consists of a central foot, clearly demarcated from the surrounding hyponota by a distinct foot groove (Fig. 7B, C). Similar to the dorsal notum, the hyponotum exhibited a perforated surface with numerous large pores (Fig. 7C). The foot region displayed prominent transverse folds embedded with pores and covered with mucus deposits (Fig. 7D and E). These mucus-coated folds are essential for facilitating locomotion and adhesion, enabling the slug to move across and adhere to various substrates in its environment.

### Histological and histochemical observations

**Fig. 8:**
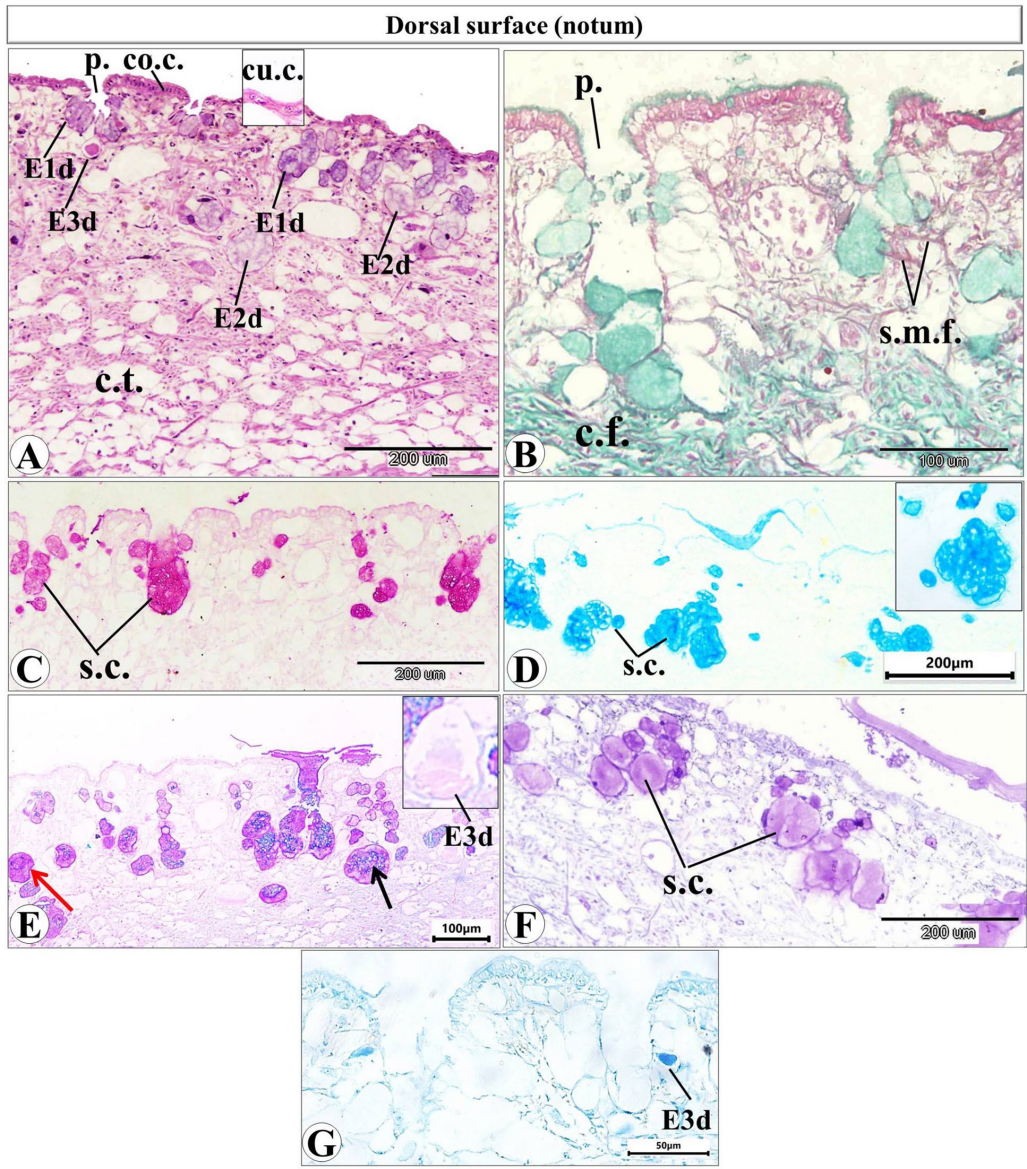
Photomicrograph of a transverse section (T.S.) of *Eleutherocaulis alte* showing the dorsal surface (notum) at 40× magnification. **(A)** H&E staining reveals the outer epithelium consists of columnar or cuboidal cells with several pits (p), the sub-epithelial layer of areolar connective tissue (C.T.). and three types of secretory cells (s.c.) (E1d, E2d, and E3d). **(B)** Masson trichrome showing the collagenous fibers (cf.) and smooth muscle fibers (s.m.f.) of the sub-epithelial layer. **(C)** PAS reaction indicates the mucin secretion of the subepithelial secretory cells (s.c). **(D)** Alcian blue (pH = 1) detects the sulphated mucosubstances in different secretory cells (s.c.). **(E)** Combined Alcian blue-PAS reaction and **(F)** toluidine blue, confirms that these secretory cells are mixed glands containing both acid and neutral mucopolysaccharides (either with large amount of acid mucopolysaccharides (black arrow) or large amount of neutral mucopolysaccharides (red arrow). **(G)** Bromophenol blue, the protein secretion appeared only in the 3rd secretory cell type (E3d).

**Fig. 9:**
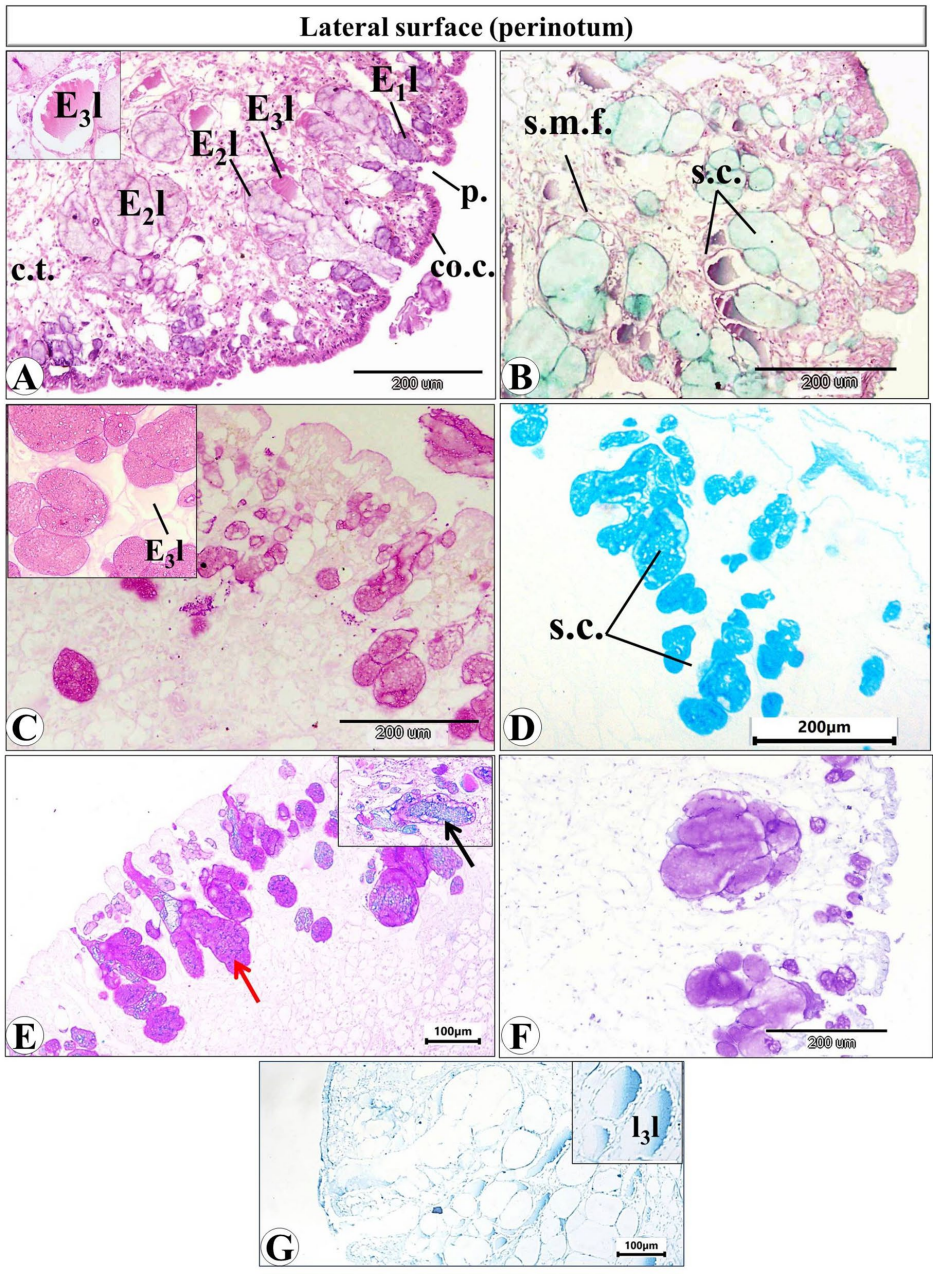
Photomicrograph of transverse section of *Eleutherocaulis alte* showing the lateral surface (perinotum) at 40× magnification. **(A)** H&E staining shows the outer epithelial columnar cells (co.c.) with several superficial pits (p) and the sub-epithelial secretory cells (s.c.) (E_1_l, E2l and E3l). **(B)** Masson trichrome showing the sub-epithelial layer of areolar connective tissue (C.T.), collagenous fibers (cf.), and smooth muscle fibers (s.m.f.). **(C)** PAS showing the highly positive reaction of E_1_l, E2l, and the faint reaction of E3l secretory cells. (**D)** Alcian blue (pH = 1) stains the sulphated mucosubstances in E_1_l, E2l. **(E)** Combined Alcian blue-PAS reaction confirms that the lateral secretory cells are mixed glands containing both acid (black arrow) and neutral mucopolysaccharides (red arrow). **(F)** Toluidine blue, showing the metachromatic structures in the secretory cells. **(G)** Bromophenol blue, which only stains in the 3rd secretory cell type (E3l).

**Fig. 10:**
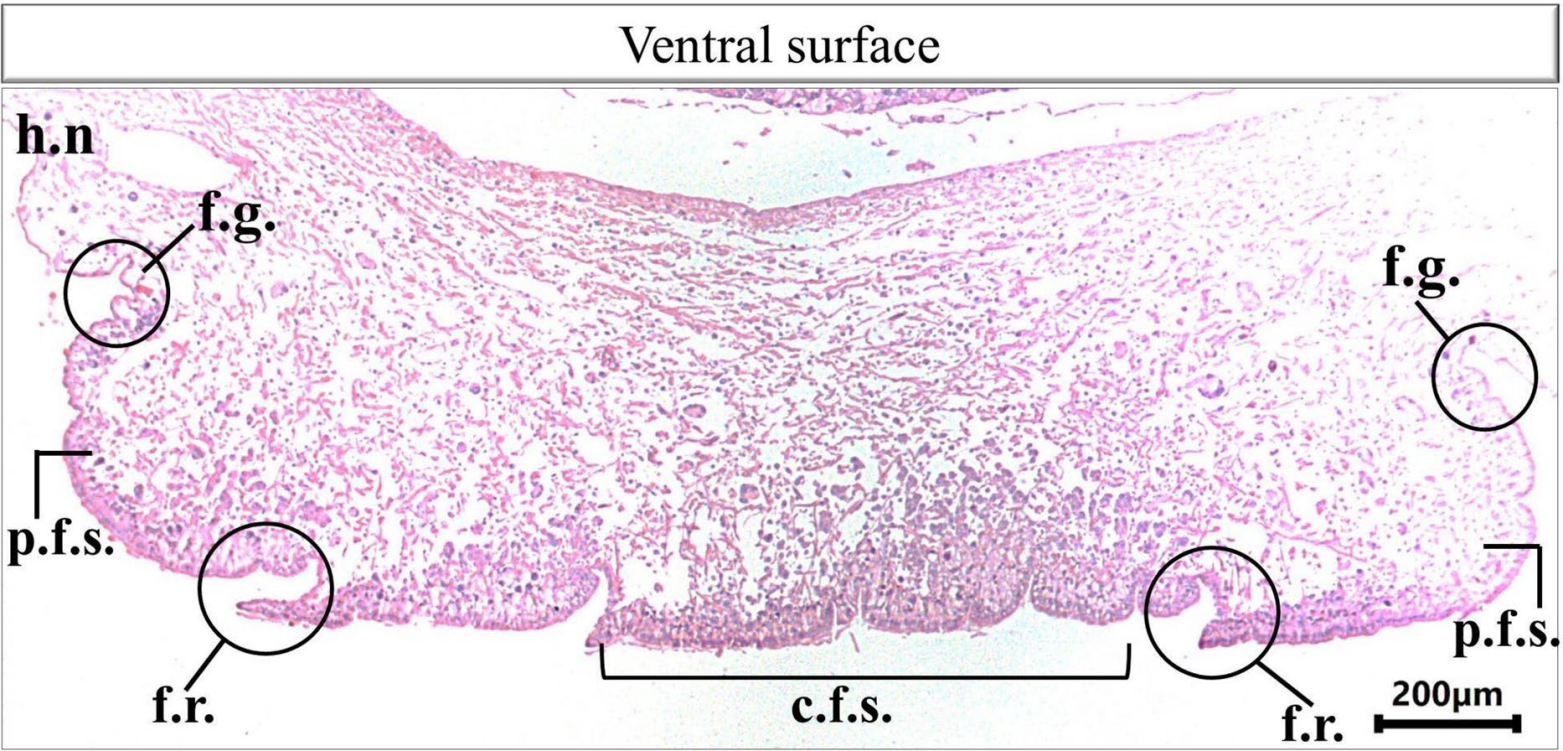
Photomicrograph of a transverse section of *E. alte* showing the ventral surface at 10× magnification. H&E staining illustrates the hyponotum (h.n.), foot groove (f.g.), the foot sole consists of peripheral foot surfaces (p.f.s.), foot ridges (f.r.) and the central foot sole (c.f.s).

**Fig. 11:**
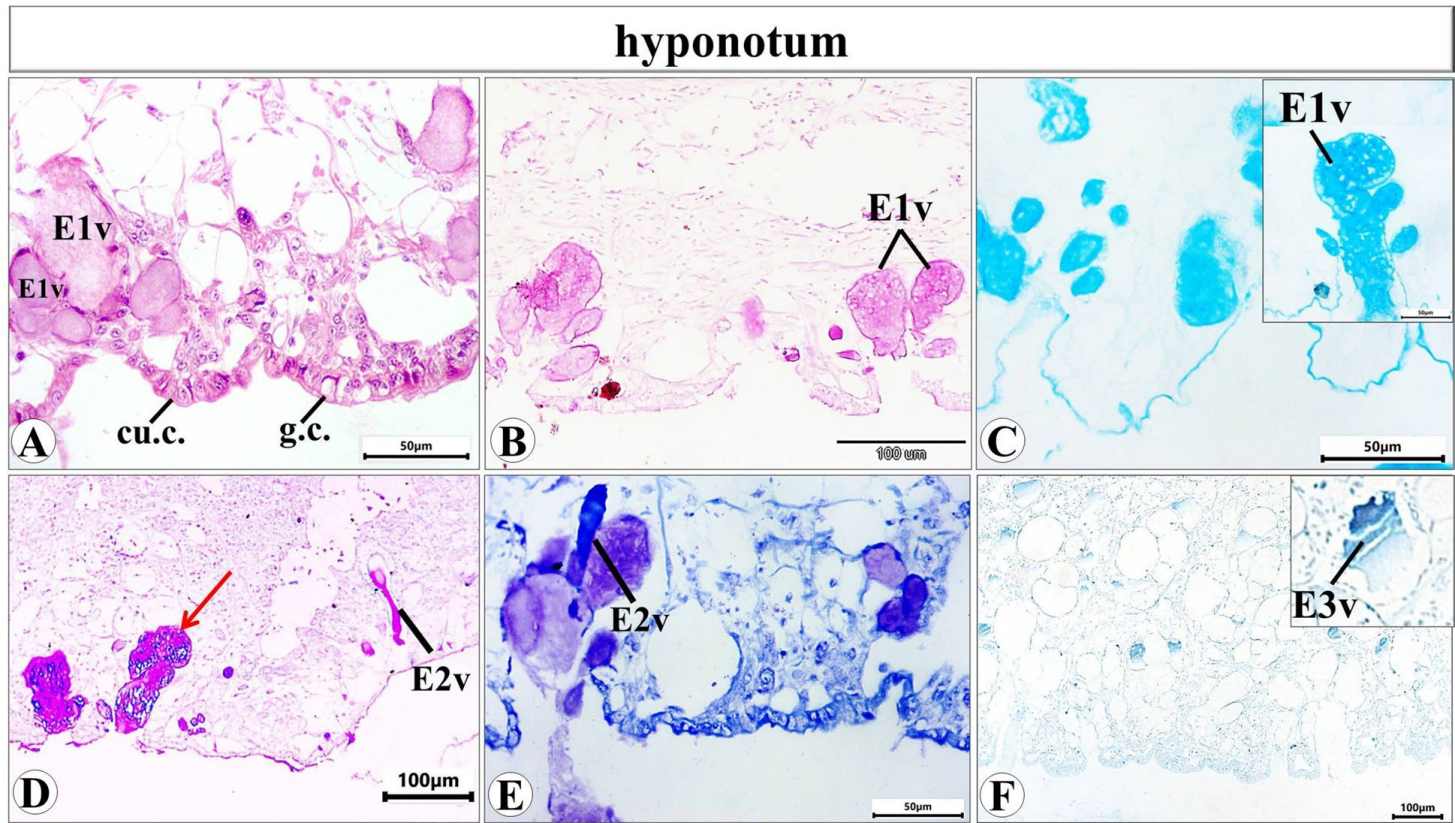
Photomicrograph of a transverse section of *E. alte* showing the hyponotum of the ventral surface with 3 types of secretory cells (E1v, E2v, and E3v) at 40× magnification. **(A)** H&E staining reveals simple cuboidal epithelial cells with goblet cells of the outer surface and the predominant sub-epithelial secretory cells (E1v). **(B)** PAS and **(C)** Alcian blue (pH = 1) show the positive reaction of E1v. **(D)** Combined Alcian blue-PAS reaction demonstrates that the E1v are mixed glands (red arrow) and E2v with neutral mucopolysaccharide contents. **(E)** Toluidine blue, showing the metachromatic contents of E1v and orthochromatic (neutral mucins) in E2v. **(F)** Bromophenol blue, showing the protein content of the 3rd secretory cell type (E3v).

**Fig. 12:**
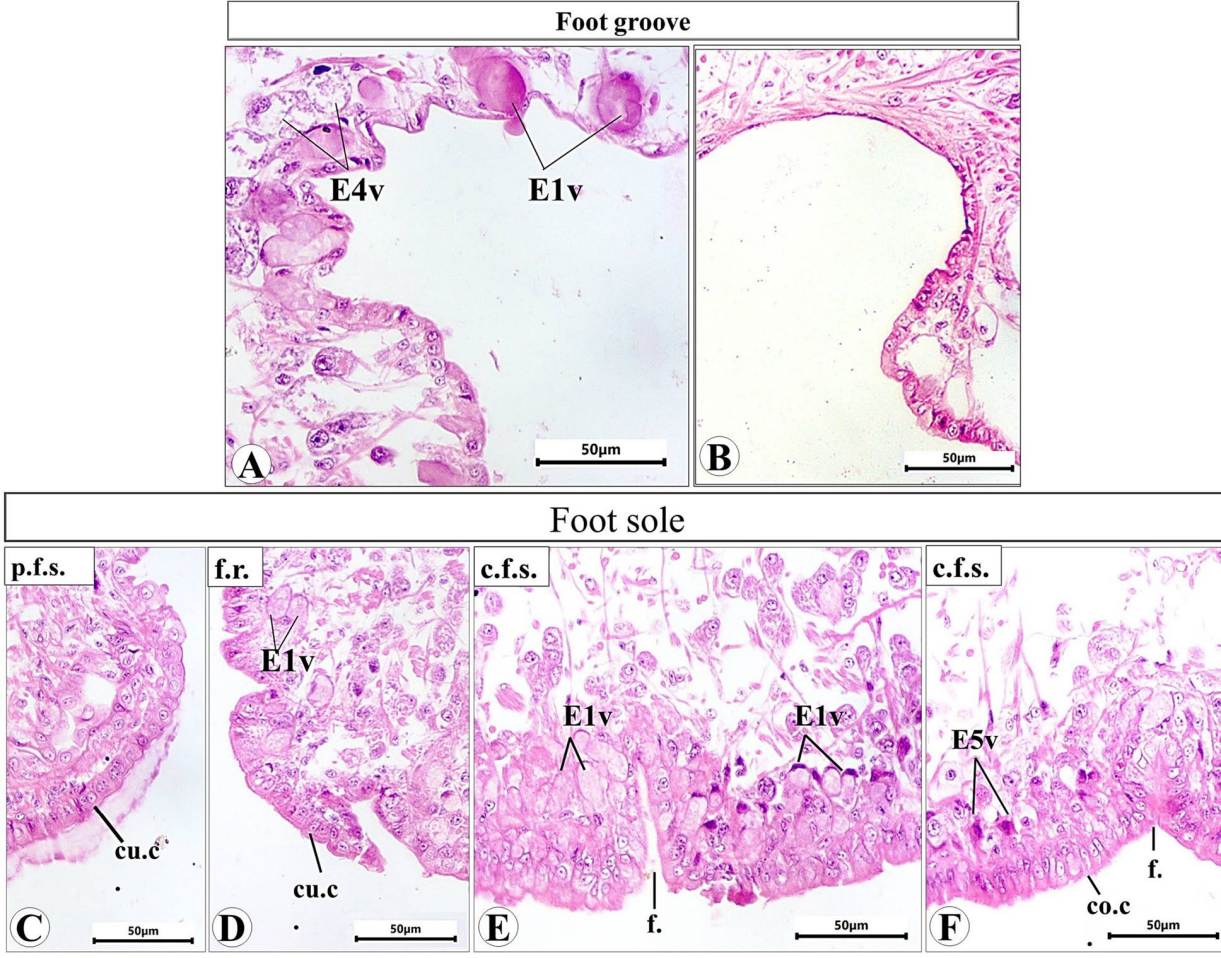
Photomicrograph of a transverse section of *E. alte* showing the foot groove and foot sole of the ventral surface at 40× magnification. H&E staining demonstrates: (A, B) the foot groove near the hyponotum containing E1v secretory cells, and grooves closer to the foot; (C) the peripheral foot surface (p.f.s.); (D) foot ridges (f.r.) consisting of cuboidal epithelial cells (cu.c.); and (E, F) the central foot sole, composed of short columnar cells (co.c.) with multiple furrows (f). The predominant secretory cell types are E1v and E4v, the latter exhibiting a stellate shape with deeply stained acidophilic cytoplasm.

**Fig. 13:**
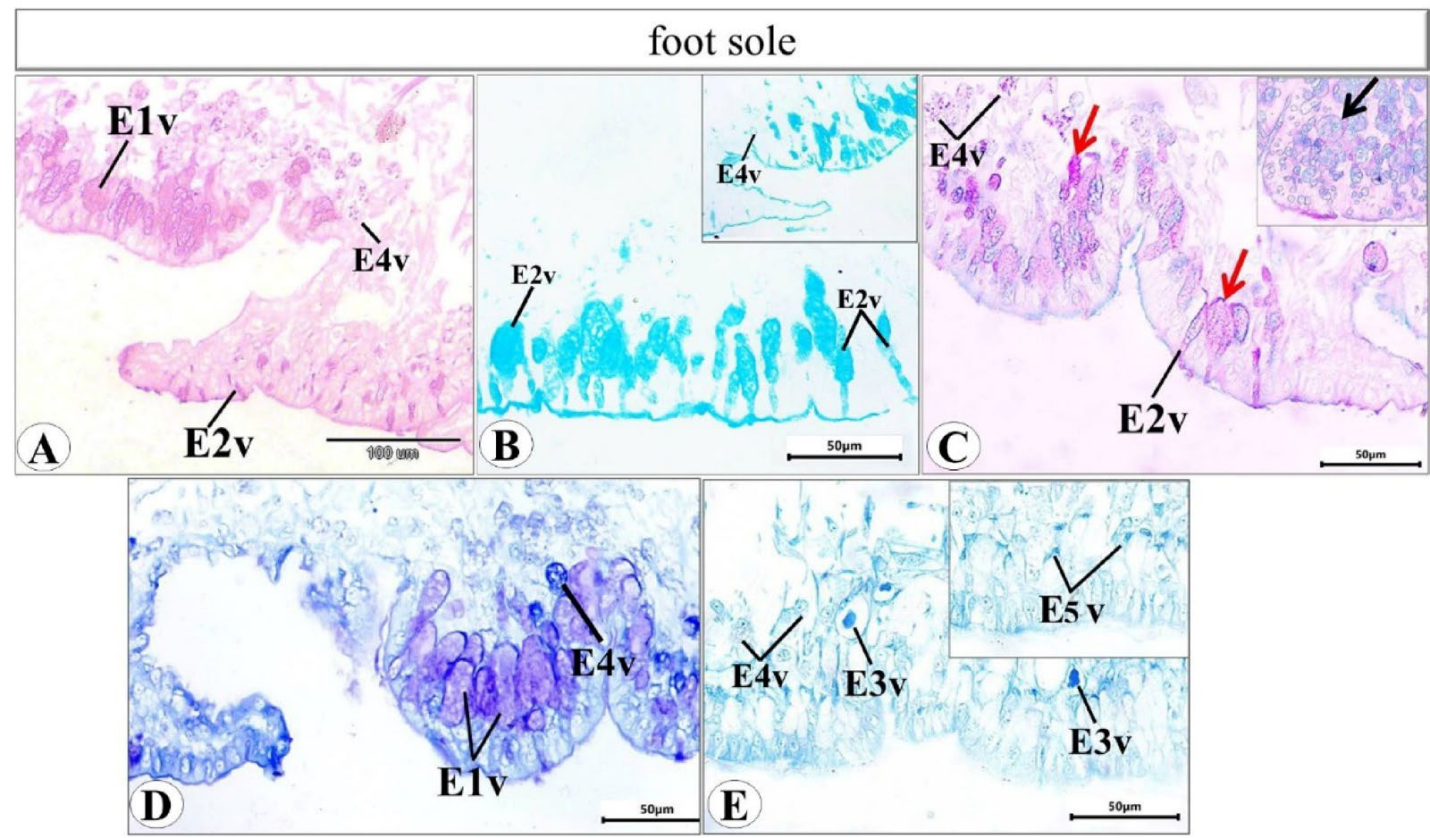
Photomicrograph of transverse section of *Eleutherocaulis alte* showing the foot sole of the ventral surface with the 4 types of secretory cells (E1v, E2v, E3v, and E4v) at 40× magnification. (**A)** PAS and (**B)** Alcian blue (pH = 1) showing the strong positive reaction of the secretory cells. (**C)** Combined Alcian blue-PAS staining confirms that these cells are mixed glands containing both acid (black arrow) and neutral mucopolysaccharides (red arrow). (**D)** Toluidine blue, showing the metachromatic contents of some secretory cells (s.c. magenta color) and orthochromatic (blue color) in others. (**F)** Bromophenol blue, showing the protein content of the 3rd (E3v) and 4th type (E4v) secretory cell type.

 Histological examination of the prepared serial transverse sections from each region (anterior, mid and posterior) of *E. alte* showed that the structure of the body surfaces and the mucus producing cells do not differ in the three regions, however the anterior region characterized by the presence of extremely short gland named as suprapedal gland (spg) that located in the body cavity above the foot. The body surface in different regions consists of epithelial (epidermis) and subepithelial layers. The epithelial cells vary in their shape from cuboidal to columnar cells (Figs. 8A and 9A). The sub-epithelial layer consists of areolar connective tissue (C.T.) with collagenous fibers and smooth muscle fibers (Figs. 8B and 9B). Different types of subepithelial mucus secretory cells were found either superficially or embedded deeply in the C.T. layer (Figs. 8, 9, 10, 11, 12 and 13). The nomenclature of these gland cells and their secretory nature were summarized in Table 1.

**Table 1:** The secretory nature of different mucus secretory cells of *Eleutherocaulis alte* in different body regions.

Body region	Dorsal surface (notum)			Lateral surface (perinotum)			Ventral surface					Suprapedal gland	
							Hyponotum + Foot groove + Foot sole						
Gland cell name	E1d	E2d	E3d	E1l	E2l	E3l	E1v	E2v	E3v	E4v	E5V	Type I	Type II
Description	oval or pear with basophilic cytoplasm	rounded with light staining basophilic vacuolated cytoplasm	rounded cells with homogenous acidophilic secretion	Similar to E1d	Similar to E2d	Similar to E3d	oval with homogenous acidophilic cytoplasm	elongated club cells???	similar to E_3_d and E_3_l	rounded cells with heterogeneous cytoplasm and fine basopilic granules.	stellate shape with deeply stained acidophilic cytoplasm	with heterogenous cytoplasm and small basophilic granules or large acidophilic secretory granules	with homogenous acidophilic cytoplasm with different degree of staining
Histochemistry													
PAS	+++	+++	+	+++	+++	+	++	++	+	+++ Cytoplasmic granules	++	+(small granules) + large granules	+++
Alcian blue (pH = 1)	+++	+++	+	+++	+++	+	++	++	+	+			
Combined PAS & Alcian blue (pH = 2.5)	Mixed +++ Magenta +++blue	Mixed +++ Magenta +++blue	+ (very faint magenta)	Mixed +++ Magenta +++blue	Mixed +++ Magenta +++blue	+ (very faint magenta)	Mixed +++ Magenta +++blue	+++ magenta	+ (very faint magenta)	+++ magenta	++ magenta	-	Mixed +++ Magenta +++ blue
Toludine blue	Metachromatic	metachromatic	metachromatic	Metachromatic	metachromatic	metachromatic	metachromatic	Orthochromatic	metachromatic	Cytoplasmic granule Orthochromatic	metachromatic	Orthochromatic	metachromatic
Bromophenole blue	-	-	+++	-	-	+++	-	-	+++	+ Cytoplasmic granules	+++	+++	-

Abbreviation: (+++) very strong reaction, (++) strong rection, (+) faint reaction, (-) negative reaction.

**In the dorsal surface (notum)**, the epithelial layer consists of columnar to cuboidal cells.

with several superficial pores (pits), the subepithelial layer possesses several secretory cells of different sizes and shapes. These cells either aggregated around and opened directly in the superficial pits or embedded deeply in the C.T. layer. They are differentiated into 3 types (E1d, E2d and E3d) that vary in their shape and size (Fig. 8A). The most prominent type is E1d, they are oval or pear in shape with basophilic cytoplasm which open into the surface either separately or aggregated to each other and open into a common superficial pit and so they considered to be the main source of the mucus that produced on the dorsal surface, The E2d, are large, rounded shape cells with light staining basophilic vacuolated cytoplasm. The E3d types are fewer in number, rounded in shape, and contain homogenous acidophilic secretion (Fig. 8A).

Histochemical staining showed that the E1d and E2d are deeply stained with PAS reaction (Fig. 8C) and Alcian blue pH = 1 (Fig. 8D). The combination of PAS and Alcian blue pH = 2.5 (Fig. 8E) showed that they are mixed glands that secrete a mixture of neutral and acidic mucopolysaccharides with different degrees of staining. The E1d and E2d cells appeared with strong positive reactions (some of them mostly contain the acidic mucopolysaccharides with few neutral mucus and vice versa), the E3d cells are also mixed glands with very faint reaction (Fig. 8E). They also showed metachromatic staining with toluidine blue (Fig. 8F). With bromophenol blue, only the E3d cells showed a positive reaction (Fig. 8G).

**The lateral surface (perinotum)** consists of columnar epithelial cells with several pits. Secretory cells are also differentiated into the same 3 types that are present in the dorsal surface; here they are named E1l, E2l, and E3l (Fig. 9). Their structures are like E1d, E2d, and E3d, respectively. The E1l and E2l are also the most predominant type in the lateral surfaces (Fig. 9A), they share their secretory nature with those of E1d, E2d (gave the same reaction with PAS (Fig. 9C), Alcian blue pH = 1 (Fig. 9D), combined PAS & Alcian blue pH = 2.5 (Fig. 9E) and the toluidine blue (Fig. 9F). The E_3_l gave a faint reaction with PAS (Fig. 9C) and a strong positive reaction with bromophenol blue (Fig. 9G).

**Ventral surface** includes the hyponotum, the foot groove, and the foot sole (Fig. 10). In the hyponotum (Fig. 11), the body surface consists of simple cuboidal epithelium with goblet cells. Secretory cells are represented by 3 types (E1v, E2v, and E3v). The E1v appears with an oval shape and homogenous acidophilic cytoplasm (Fig. 11A) that is also described as a mixed gland with the histochemical examination (Fig. 11B-D). The E2v are elongated thin cells, secret neutral mucopolysaccharides which are demonstrated by the combined PAS & Alcian blue pH = 2.5 reaction (Fig. 11D) and confirmed by the metachromatic dye (toluidine blue) as they possess orthochromatic structure (neutral mucins) (Fig. 11E). The 3rd type (E3v) is also similar in their structure to E3d and E3l, they possess a protein secretion (Fig. 11F).

The foot groove can be differentiated into two 2 halves, one of them that present close to the hyponotum (Fig. 12A) and the other close to the foot sole with epithelial cells vary from cuboidal to squamous (Fig. 12B). Two types of secretory cells (E1v and E4v) were noticed in the side that are close to the hyponotum (Fig. 12A). E1v is the same type that previously described in the hyponotum. E4v is a type with rounded to oval shape and heterogeneous cytoplasm containing fine basophilic granules.

The foot sole can be differentiated into 3 regions (peripheral foot surfaces, foot ridges and the central foot sole) in histological sections according to the shape of epithelial cells, type and distribution of secretory cells (Fig. 12C-E).

The peripheral foot surface and the foot ridges consist of cuboidal epithelial cells (Fig. 12C and D). The central foot sole (Fig. 12E and F) consists of columnar epithelial cells with basal nuclei. The most predominant cell types are E1v (like that of the hyponotum and the E5v (stellate shape with deeply stained acidophilic cytoplasm).

Histochemical techniques revealed different secretory cells in the foot sole of ventral surface take the same manner as that of hyponotum with PAS (Fig. 13A), Alcian blue pH = 1 (Fig. 13B), combined PAS & Alcian blue pH = 2.5 (Fig. 13C), toluidine blue (Fig. 13D), and bromophenol blue (Fig. 13E). E5v showed a positive reaction with bromophenol.

### Suprapedal gland

**Fig. 14:**
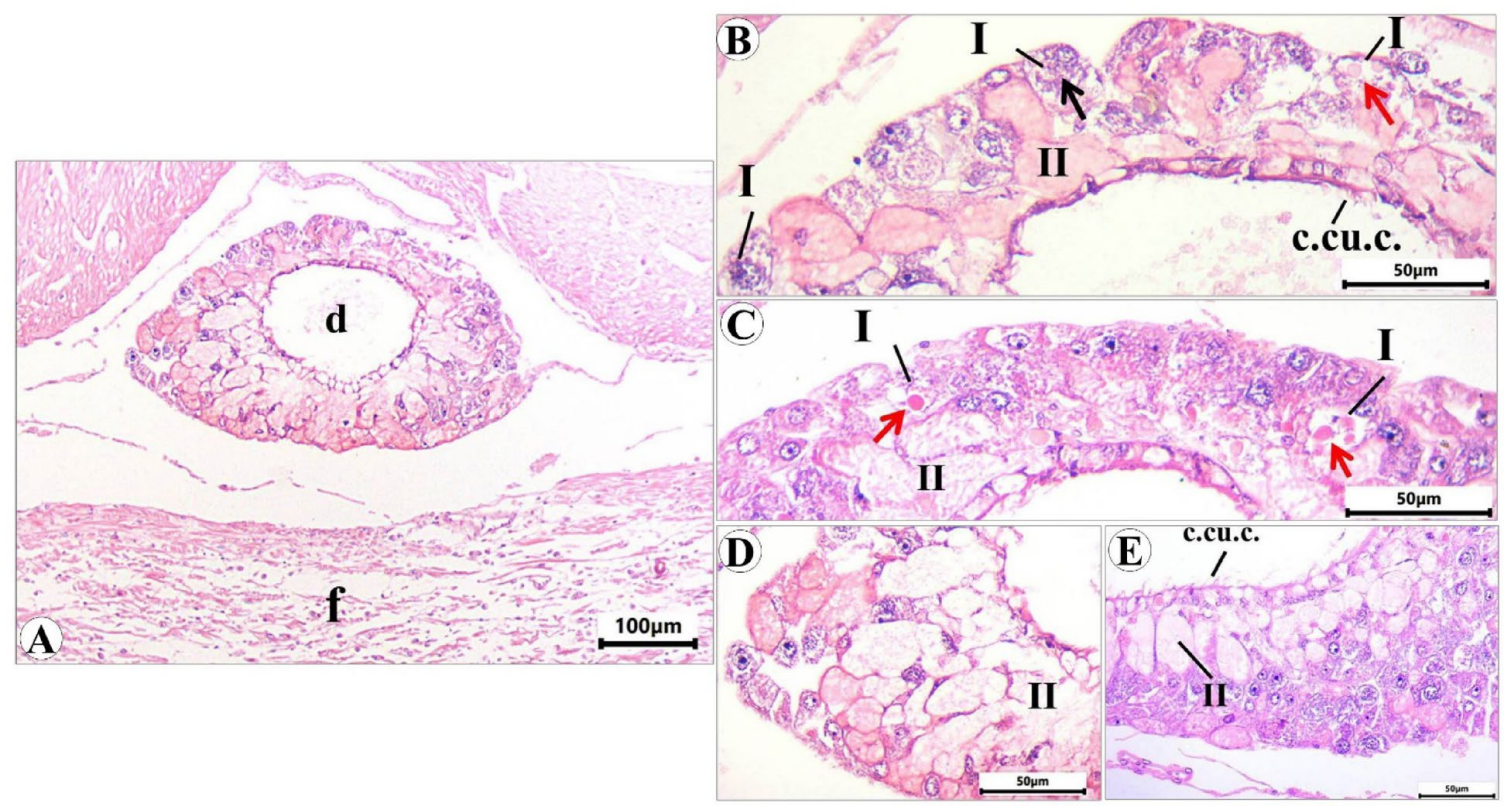
Photomicrograph of transverse sections of *E. alte* showing the suprapedal gland. (A) At 10× magnification, H&E staining demonstrates the gland located in the body cavity above the foot (f), with secretory cells releasing their products into the central duct (d), which is lined by ciliated cuboidal epithelium with goblet cells. (B–E) At 40× magnification, two secretory cell types are observed: Type I cells display heterogeneous cytoplasm containing fine basophilic granules (black arrow) or large acidophilic granules (red arrow); Type II cells constitute the bulk of the gland, located adjacent to the duct, with acidophilic cytoplasm exhibiting variable staining intensity.

It is a short tubular gland located in the cavity of the body above the midline of the foot sole. Examination of serial sections revealed that this gland restricted only to the anterior region of the body (Fig. 14A). It consists of two main types of unicellular secretory cells that flow their secretion (via merocrine secretion) into the central duct which is lined with ciliated cuboidal epithelium with goblet cells. The 1 st type (I), present away from the duct with heterogeneous cytoplasm containing fine basophilic granules (Fig. 14B) and/or large acidophilic secretory granules (Fig. 14C). The 2nd type (II) represents the main bulk of the gland and is presented close to the duct that appears with homogenous acidophilic cytoplasm with different degrees of staining (Fig. 14C and D).

**Fig. 15:**
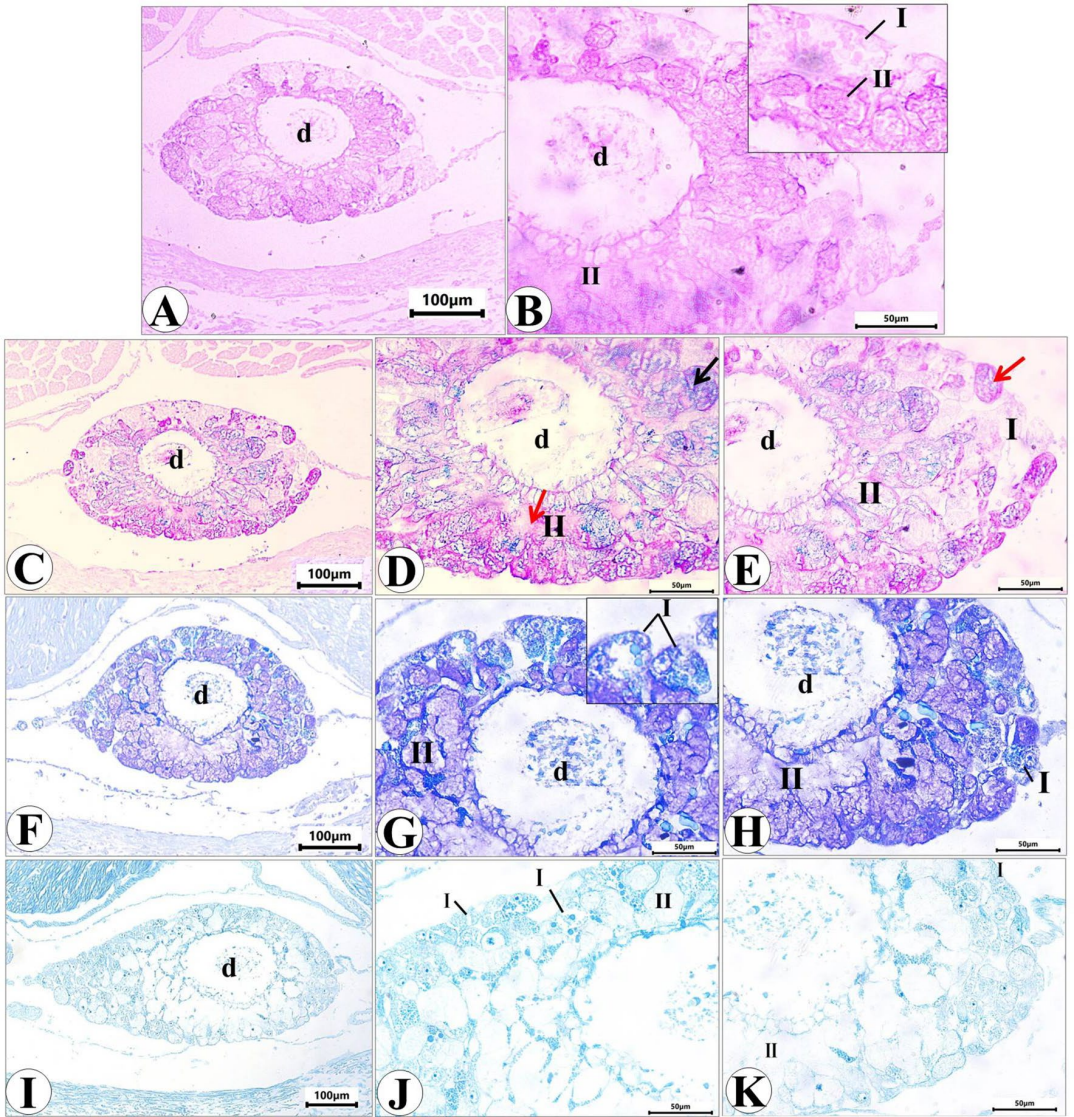
Photomicrograph T.S. of *Eleutherocaulis alte showing* the suprapedal gland stained with: (**A)** and **(B)** PAS showing the highly positive reaction of type II only. (**C-E)** and **(F-H)** Combined PAS & Alcian blue and toluidine blue, respectively, showing that type I secretes neutral mucopolysaccharides (orthochromatic) and type II are mixed glands that secrete acid mucopolysaccharides (metachromatic). (**I-K)** The bromophenol blue showed that the protein secretion was restricted to type I of the gland.

The mucin secretion in this gland stained intensely with PAS reaction in type II secretory cells however, that in type I showed a very weak reaction (Fig. 15A and B). Combined PAS & Alcian blue pH = 2.5 showed very strong reaction in type II, indicating that they are mixed glands, most of them secrete acid mucopolysaccharides & weak neutral, and vice versa in the others. However, the type I secretes very weak neutral mucopolysaccharides (Fig. 15C-E). The metachromatic dye (toluidine blue) also confirms the presence of orthochromatic substances (neutral mucopolysaccharides) in type I of secretory cells and metachromatic substances (acid mucopolysaccharides) in type II (Fig. 15F-H). The bromophenol blue stain showed that the protein secretion is restricted to the type I gland (Fig. 15I-K).

### Antimicrobial activity

The antimicrobial potential of the mucus from *E. alte* was assessed against different pathogenic bacterial and fungal strains using the agar well diffusion method.

Statistical comparison revealed that crude mucus (10 mg/mL) produced significantly larger inhibition zones than the positive controls, gentamicin (GM) for bacteria and fluconazole (FLZ) for fungi, with the exception of *A. niger*, which exhibited complete resistance to the mucus extract. Levels of significance were indicated as *P* < 0.05 (*), *P* < 0.01 (**), and *P* < 0.001 (***), highlighting the strong inhibitory activity of the mucus against most tested pathogens.

**Fig. 16:**
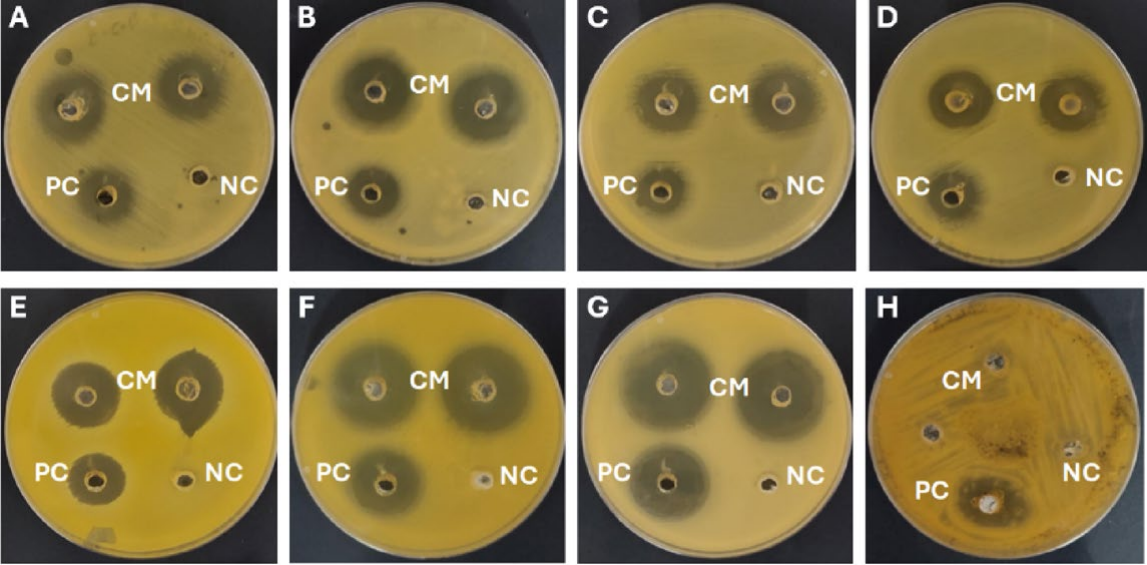
The inhibition zone (mm) of crude mucus (CM) of *Eleutherocaulis alte* at a concentration of 10 mg/ml against (**A**) *E. coli* (**B**) *K. pneumoniae*, (**C**) *S. typhi*, (**D**) *S. epidermidis*, (**E**) *S. aureus*, (**F**) *B. subtilis*, (**G**) *C. albicans*, and (**H**) *A. niger*. PC: Fluconazole and Gentamicin at a concentration of 10 mg/ml (positive control); NC: 10% DMSO (negative control).

As shown in Table 2; Fig. 16, the crude mucus demonstrated varying degrees of inhibition across all susceptible strains, with the largest zone of inhibition observed against *C. albicans* (29.50 ± 0.71 mm), followed by *B. subtilis* (28.00 ± 0.00 mm), and *S. aureus* (22.00 ± 1.41 mm). The mucus showed moderate activity against *E. coli* (21.50 ± 0.71 mm), *K. pneumoniae* (19.00 ± 1.41 mm), *S. epidermidis* (19.50 ± 0.71 mm), and *S. typhi* (18.50 ± 0.71 mm). No inhibitory effect was observed against *A. niger*. When compared to the positive control, gentamicin (GM, 10 mg/mL), the mucus exhibited superior antimicrobial activity across all bacterial strains. Similarly, its antifungal effect against *C. albicans* exceeded that of fluconazole (FLZ, 10 mg/mL), with inhibition zones of 29.50 ± 0.71 mm and 23.00 ± 1.00 mm, respectively.

**Table 2:** Antimicrobial activity (determined as Inhibition zone diameter) of the crude mucus (CM) from *Eleutherocaulis alte*. Values represent the mean ± standard deviation (SD) of triplicate measurements. Asterisks indicate statistically significant differences between crude mucus and the corresponding positive control (gentamicin for bacteria; fluconazole for fungi): *P* < 0.05 (*), *P* < 0.01 (**), and *P* < 0.001 (***). GM: Gentamicin; FLZ: Fluconazole. NA: no activity detected.

Treatment	Diameter of inhibition zone (mm)							
	*E. coli*	*S. typhi*	*K. pneumoniae*	*B. subtilis*	*S. aureus*	*S. epidermidis*	*C. albicans*	*A. niger*
CM (10 mg/ml)	21.50 ± 0.71^**^	18.50 ± 0.71^*^	19.00 ± 1.41^*^	28.00 ± 0.00^***^	22.00 ± 1.41^**^	19.50 ± 0.71^*^	29.50 ± 0.71^**^	NA
GM	16.00 ± 0.00	13.33 ± 1.52	14.33 ± 1.52	20.66 ± 0.57	16.66 ± 0.57	15.33 ± 1.15		
FLZ							23.00 ± 1.00	17.66 ± 0.57^***^

MIC, MBC, and MFC values for the mucus were determined using the broth microdilution method (Table 3). The mucus demonstrated potent activity against *C. albicans*, with the lowest MIC and MFC values recorded at 3.9 µg/mL and 7.8 µg/mL, respectively. Among bacterial strains, *B. subtilis* was the most sensitive, with an MIC of 7.8 µg/mL and an MBC of 15.62 µg/mL. *S. aureus* also showed considerable susceptibility (MIC: 15.62 µg/mL; MBC: 31.25 µg/mL). Moderate activity was observed against *E. coli* (MIC: 31.25 µg/mL; MBC: 62.5 µg/mL) and *K. pneumoniae* (MIC and MBC: 62.5 µg/mL). *S. typhi* was the least sensitive bacterial strain tested, with an MIC of 125 µg/mL and an MBC of 500 µg/mL. In contrast, *A. niger* showed no inhibition and was therefore excluded from subsequent MIC, MBC, and MFC determinations, as its weak inhibition in the preliminary assay indicated that reliable quantitative evaluation would not be meaningful.

**Table 3:** Minimum inhibitory, minimum bactericidal, and minimum fungicidal concentrations of crude mucus from *Eleutherocaulis alte* against different human pathogens.

Target pathogens	Mucus concentration (µg/mL)	
	MIC	MBC or MFC
*E. coli*	31.25	62.5
*S. typhi*	125	500
*K. pneumoniae*	62.5	62.5
*B. subtilis*	7.8	15.62
*S. aureus*	15.62	31.25
*S. epidermidis*	62.5	62.5
*C. albicans*	3.9	7.8

MIC, Minimum Inhibitory Concentration; MBC, Minimum Bactericidal Concentration; MFC, Minimum Fungicidal Concentration.

Collectively, these results suggest that the mucus possesses broad-spectrum antimicrobial activity, with particular effectiveness against Gram-positive bacteria and *C. albicans*, while showing limited or no activity against *A. niger*.

## Discussion

In recent years, considerable efforts have been devoted to surveying invasive gastropod pests in Egyptian agricultural fields^46–50^. Within the family Veronicellidae, a group of phytophagous slugs primarily inhabiting tropical and subtropical regions of Asia, America, and Africa^51–54^, species are known for their hermaphroditic nature and lack of internal shells^55–57^. The present study documents the occurrence of the leatherleaf slug *E. alte* in various agricultural fields and ornamental plants at the Assiut University Farm, Egypt. To confirm species identification, the mitochondrial cytochrome c oxidase I (COI) gene, commonly used in DNA barcoding^58^, was sequenced. The COI-based results corresponded closely with multiple *E. alte* sequences deposited in GenBank, strengthening the reliability of the species identification.

From a taxonomic standpoint, Sajan et al.^59^ recognized *Laevicaulis* (Simroth, 1913) as the senior synonym of *Eleutherocaulis* (Simroth, 1913), and consequently treated *Vaginulus comorensis* (Fischer, 1883) as a junior synonym of *Vaginulus alte* (Férussac, 1822). However, the systematics of *Laevicaulis* remain unresolved, primarily due to the dependence on variable external morphological traits, such as coloration and body size, for species differentiation. Subspecies distinctions within *Laevicaulis* have been particularly discussed. Simroth^60^ highlighted similarities between *L. striatus* and *L. stuhlmanni*, especially in female genitalia, noting differences mainly in phallic morphology. In *L. stuhlmanni*, the phallus is cylindrical with a distal flattened disc, while in *L. striatus*, a subdistal swelling (“collar”) is evident. Observations of *Laevicaulis alte* from the present study align with Bishop^31^, who also described a subdistal phallic swelling. Liberto et al.^11^ classified Libyan *Laevicaulis* populations as *L. striatus*, yet previous findings by Colosi^61^ and Forcart^62^ suggested variability in phallus morphology within *L. stuhlmanni* and *L. striatus*, cautioning against synonymizing the two. Recent reviews call for molecular analyses to clarify the taxonomic relationships among *Laevicaulis* species, including *E. striatus* as suggested by Liberto et al.^11^. Morphologically, Egyptian *Laevicaulis* populations display similarities to those in Libya, although some differences exist, such as phallus stalk length and notum coloration^62^. Populations from Egypt exhibit a reddish-brown dorsal notum with a median stripe and lateral spots, consistent with the features described for *L. striatus*. Conversely, studies by Ali and Robinson^9,10^ on *Laevicaulis alte* from Abo Rawash highlighted distinctions such as a darker dorsal notum with a central white line and grey to creamy hyponotal coloration.

The present findings, based on both morphological and molecular evidence, confirm the identity of the studied population as *E. alte* (Family: Veronicellidae). From a histological perspective, terrestrial slug epidermis serves multiple roles, including respiration, osmoregulation, locomotion, and defense, aided by a protective mucus layer^63^. Consistent with previous studies on terrestrial gastropods^17,40,63–67^, the epidermis of *E. alte* comprises epithelial and subepithelial connective tissue layers. Comparative analysis with *Veronicella floridana*^66^ revealed similarities in epidermal architecture, with cuboidal to columnar epithelial cells predominating, except in transitional regions where squamous epithelium is present. Mucus-secreting cells were found interspersed among epidermal cells and embedded within the connective tissue. Detailed histological examination identified seven mucus cell types in *E. alte*, grouped according to their distribution in the dorsal notum, lateral perinotum, and ventral surfaces. Comparisons with *V. floridana* revealed analogous secretory cell types^66^, despite differences in the number and characteristics of gland types. Notably, the suprapedal gland in *E. alte*, a short tubular structure consisting of Type I and Type II cells, parallels the V1 and V2 cell types described in *V. floridana*. Histochemical analyses demonstrated that Type II cells in *E. alte* predominantly secrete a mix of acidic and neutral mucopolysaccharides, while Type I cells produce weak neutral substances, a pattern comparable to that seen in *V. floridana*. Mucus secretion is critical not only for physiological functions but also for its bioactive properties. Previous research has established the therapeutic potential of snail mucus, including antimicrobial activities^68^, warranting further investigation.

The antimicrobial assessment of *E. alte* mucus revealed broad-spectrum activity against Gram-positive and Gram-negative bacteria, as well as *C. albicans*. These findings are supported by both the agar well diffusion and broth microdilution assays, which demonstrated large inhibition zones (29.50 and 28.00 mm) and remarkably low MIC values (3.9 and 7.8 µg/mL) for *C. albicans* and *B. subtilis*, respectively. The mucus also outperformed conventional antimicrobials, gentamicin and fluconazole, when tested at equivalent concentrations, highlighting its therapeutic potential. Notably, *A. niger* was an exception, as no inhibitory effect was observed against this fungus in the agar well diffusion assay, consistent with its exclusion from subsequent MIC, MBC, and MFC determinations.

Statistical analysis further confirmed these observations, showing significant inhibitory effects of the mucus against all tested microorganisms except *(A) niger*. The highest level of significance was recorded for *(B) subtilis* (*P* < 0.001), followed by *(C) albicans* (*P* < 0.01). The consistently larger inhibition zones produced by the mucus compared to the positive controls suggest the presence of potent bioactive components with superior efficacy against these pathogens.

The potent activity against Gram-positive bacteria aligns with earlier findings on gastropod mucus, where bioactive compounds such as antimicrobial peptides (AMPs), glycoproteins, and enzymes are thought to compromise microbial membrane integrity or interfere with essential metabolic pathways^17,24^. The greater susceptibility of Gram-positive strains compared to Gram-negative bacteria could be attributed to structural differences in the bacterial cell envelope, where the outer membrane of Gram-negative organisms often acts as a barrier to many antimicrobial agents^69^. Interestingly, the mucus exhibited stronger antifungal activity against *C. albicans* than fluconazole, a commonly used antifungal drug. This supports recent studies suggesting that molluscan secretions contain unique bioactive molecules such as achacin, hyaluronic acid, and glycosaminoglycans that possess not only antibacterial but also antifungal properties^26,27^. The low MIC and MFC values (3.9 µg/mL and 7.8 µg/mL, respectively) confirm the mucus’s fungicidal effect and indicate its potential as an alternative treatment for fungal infections, especially in an era of rising antifungal resistance. While the mucus showed moderate activity against *E. coli*, *K. pneumoniae*, and *S. typhi*, the latter exhibited the highest resistance (MIC: 125 µg/mL; MBC: 500 µg/mL). This variability in effectiveness may relate to strain-specific defense mechanisms or differences in the bioavailability of mucus components. Previous work has highlighted similar variation in antimicrobial responses among bacteria exposed to slug or snail mucus^28^. From a biomedical perspective, the potent activity of *E. alte* mucus against both bacterial and fungal pathogens, especially at low effective concentrations, strongly supports its development as a natural antimicrobial agent.

The antimicrobial activity observed in *E. alte* mucus is consistent with previous findings on other terrestrial gastropods, whose mucus serves as a multifunctional barrier with both protective and therapeutic properties. The mucus secreted from the snail’s foot plays an essential defensive role, forming a hydrated layer that not only minimizes friction and prevents desiccation but also protects the animal from microbial invasion on contaminated surfaces. This ecological function is supported by its rich biochemical composition, which includes antimicrobial peptides (AMPs), glycoproteins, glycosaminoglycans (GAGs), and lectins, all of which contribute to its antimicrobial potency^25,26,70,71^.

The antibacterial effects of snail mucus have been linked to bioactive proteins such as achacin, Achatina cysteine-rich proteins (CRPs), and Mytimacin-AF, which display strong inhibitory activity against both Gram-positive and Gram-negative bacteria, including multidrug-resistant strains like *S. aureus* and *Pseudomonas aeruginosa*^72–74^. These compounds act primarily by disrupting bacterial membranes, causing leakage of intracellular contents and interfering with vital metabolic enzymes. The positively charged AMPs bind electrostatically to negatively charged microbial membranes, forming pores and ultimately leading to cell death. Gram-positive bacteria such as *B. subtilis* and *S. aureus* tend to be more susceptible because their single peptidoglycan layer is more accessible to these molecules than the dual-layered envelope of Gram-negative bacteria.

In addition to antibacterial effects, antifungal activity of mucus has been documented in multiple snail species, with reports of growth inhibition against *C. albicans*, *Aspergillus fumigatus*, *Penicillium chrysogenum*, and *Mucor racemosus*^75^. This is largely mediated by lectins and antimicrobial peptides that bind to fungal cell wall carbohydrates, disrupting membrane integrity and interfering with vital metabolic processes^76^. The high glycoprotein and mucin content in the secretion may also enhance host immune defense mechanisms against fungal pathogens.

Collectively, these findings indicate that the potent antimicrobial effects observed in *E. alte* mucus, particularly its strong inhibition of *B. subtilis* and *C. albicans*, likely result from a synergistic combination of structurally diverse antimicrobial peptides, glycoproteins, and lectins that act through membrane disruption, metabolic inhibition, and immunomodulatory mechanisms. This mechanistic understanding reinforces the biomedical relevance of *E. alte* mucus as a promising natural source for developing new antimicrobial and antifungal therapies, particularly against drug-resistant pathogens.

Comparative studies further support the relative potency of *E. alte* mucus. Trapella et al.^77^ reported broad antimicrobial activity in *Helix aspersa* mucus, whereas El-Zawawy et al.^25^ found that *Eobania desertorum* mucus exhibited stronger antibacterial effects than *H. aspersa* against drug-resistant wound pathogens. Notably, neither study reported significant inhibition of *K. pneumoniae*, whereas our findings show clear susceptibility of this strain to *E. alte* mucus. Conversely, fungal strains examined in those studies were sensitive to mucus extracts, while *A. niger* in the present work was highly resistant and showed no response to the crude mucus.

Despite the promising antimicrobial activity observed in this study, several limitations should be acknowledged. First, the specific bioactive constituents responsible for these effects remain uncharacterized, as the study did not include biochemical profiling or purification of individual compounds from the mucus. Second, the biological assays including antimicrobial were conducted entirely in vitro; thus, in vivo validation of the observed activities is necessary to confirm efficacy and safety in a physiological context. Future research should aim to isolate and identify active molecules through advanced analytical techniques, such as HPLC, LC-MS, or proteomic approaches. Additionally, animal models could be employed to evaluate the pharmacological effects and potential toxicity of mucus-derived bioactives in vivo. Overall, this integrative study offers valuable insights into the taxonomy, histology, and biomedical relevance of *E. alte*, underscoring its potential role in developing new antimicrobial therapies.

## Conclusion

This study provides an integrative investigation of *E. alte* in Assiut Governorate, Egypt, encompassing morphological, molecular, histological, and preliminary antimicrobial evaluations. Molecular data confirmed the species’ identity, while histological observations revealed mucus-secretory cells with diverse structural and chemical features. Importantly, the crude mucus of *E. alte* exhibited broad-spectrum antimicrobial activity, particularly against *B. subtilis* and *C. albicans*. These findings establish a valuable taxonomic and physiological baseline for the species and highlight its crude mucus as a promising source of natural antimicrobial agents.

Future research should focus on characterizing the chemical composition of the mucus to identify the bioactive constituents underlying its antimicrobial activity. Advanced analytical techniques such as LC–MS and HPLC profiling will be instrumental in this regard. In addition, exploring the potential synergistic effects of the mucus with standard antibiotics could enhance its translational relevance, especially in combating multidrug-resistant pathogens. Collectively, such in-depth studies, including chemical profiling, compound isolation, and in vivo assessments, are essential to fully substantiate the biomedical potential of *E. alte*.

## Supplementary Information

Below is the link to the electronic supplementary material.

Supplementary Material 1

## Data Availability

All data analyzed or generated during the research are included in the article [and its supplementary information files]. The COI sequence of *E. alte* **was uploaded to GenBank database as OR162029.**https://www.ncbi.nlm.nih.gov/nucleotide/OR162029.1?report=genbank&log$=nucltop&blast_rank=1&RID=238YCC41013.
