# Supplementary material for: Integrated morphological, molecular, histological, and antimicrobial analysis of the leather leaf slug Eleutherocaulis alte from Assiut Governorate, Egypt
Source: Sci Rep. 2026 Jan 6;16:794. doi: 10.1038/s41598-025-32703-6 (PMC12779638; doi:10.1038/s41598-025-32703-6)
Supplement: Supplementary file 1 — Supplementary Material 1 [file 41598_2025_32703_MOESM1_ESM.pdf]

Comprehensive study of leather leaf slug *Eleutherocaulis alte* (Family: Veronicellidae) from Assiut Governorate, Egypt: morphology, molecular identification, histological structure of mucus secretory cells, and potential antimicrobial effect

Authors and affiliations:

Safaa M. Ali[1], Torkia A. Mohammed[1], Shimaa H. Salem [2]*, Hayam A. Saber[1] and Asmaa R. Abdel-Malek[1]

[1] Zoology and Entomology Department, Faculty of Science, Assiut University, Assiut 71526, Egypt

[2] Botany and Microbiology Department, Faculty of Science, Assiut University, Assiut 71526, Egypt

* Corresponding author at Department of Botany and Microbiology, Faculty of Science, Assiut University, Assiut, Egypt 71526. Tel: +201067657884

E-mail: shimaa.hassan@aun.edu.eg (Shimaa H. Salem, PhD).

ORCID ID: 0000-0002-2965-8143

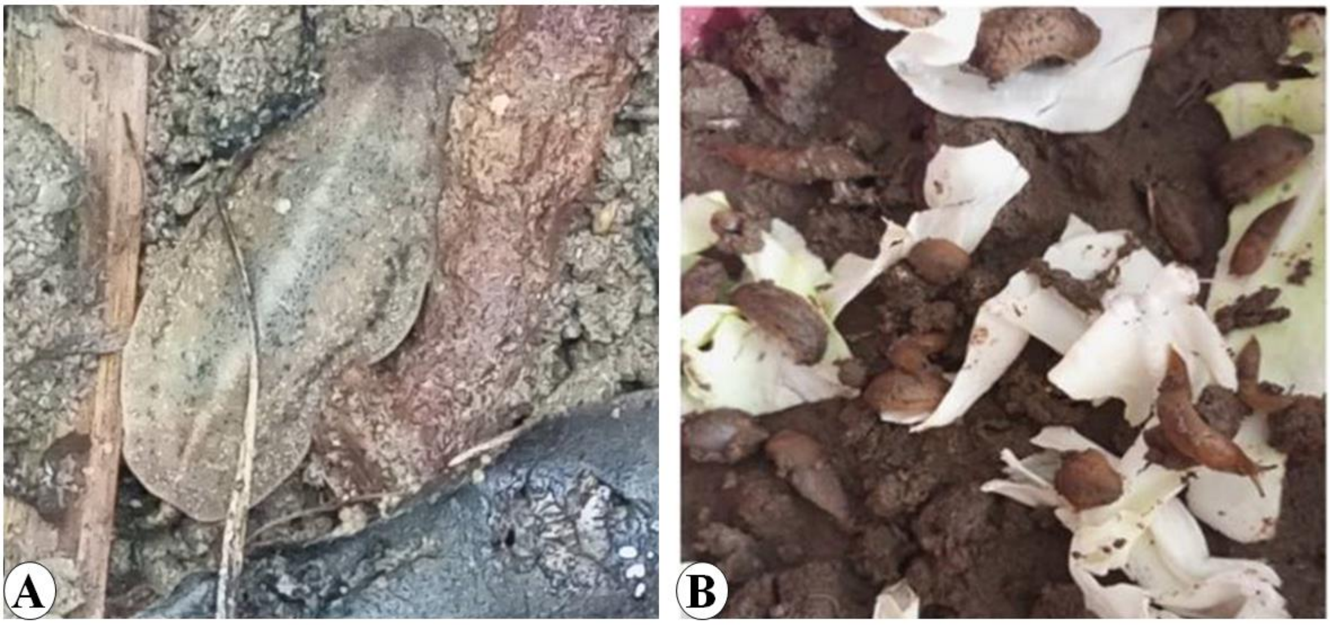

Fig. S1. A) Collection of the slug *"Eletherocaulis alte"* from the field**, B)** Slug's rearing in the laboratory.

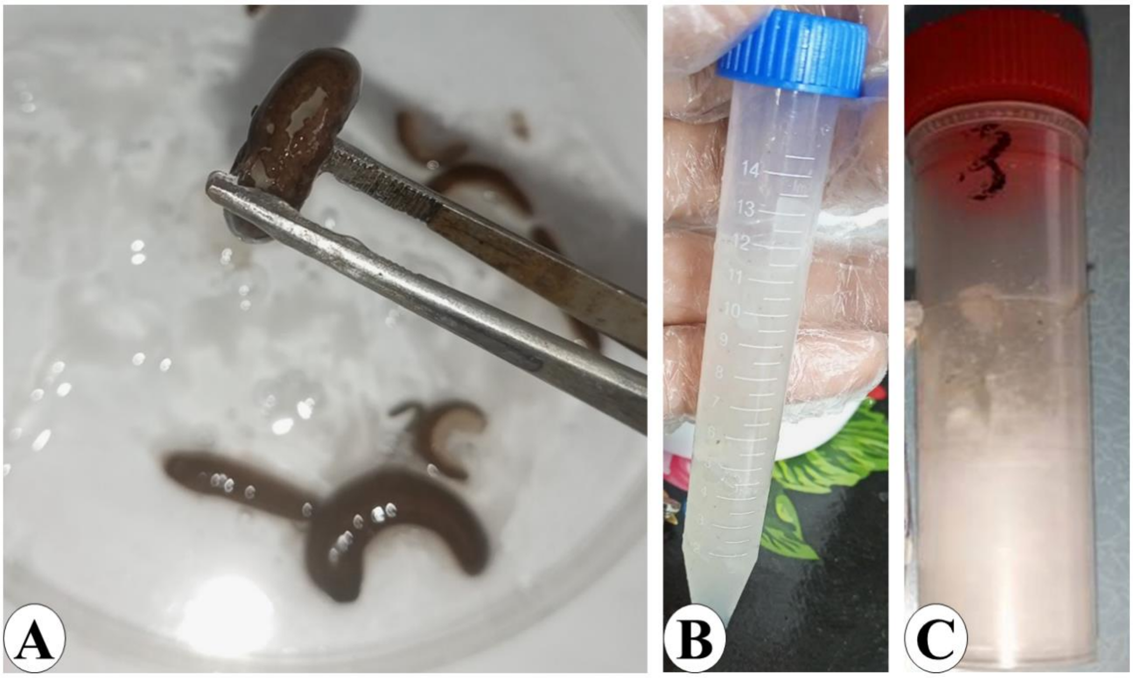

Fig. S2. A) Stimulation of the slugs to excrete the mucus, **B)** mucus collection, **C)** Lyophilized powder of the mucus.
